# Supplementary material for: The ATR inhibitor ceralasertib potentiates cancer checkpoint immunotherapy by regulating the tumor microenvironment
Source: Nat Commun. 2024 Feb 24;15:1700. doi: 10.1038/s41467-024-45996-4 (PMC10894296; doi:10.1038/s41467-024-45996-4)
Supplement: Supplementary file 1 — Supplementary Information [file 41467_2024_45996_MOESM1_ESM.pdf]

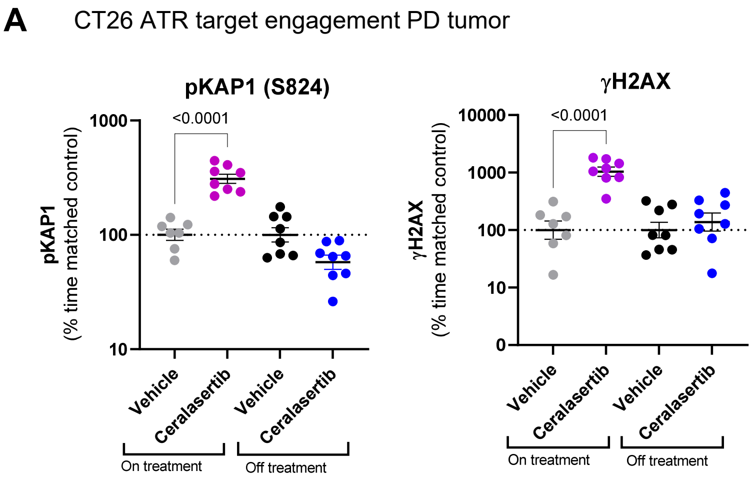

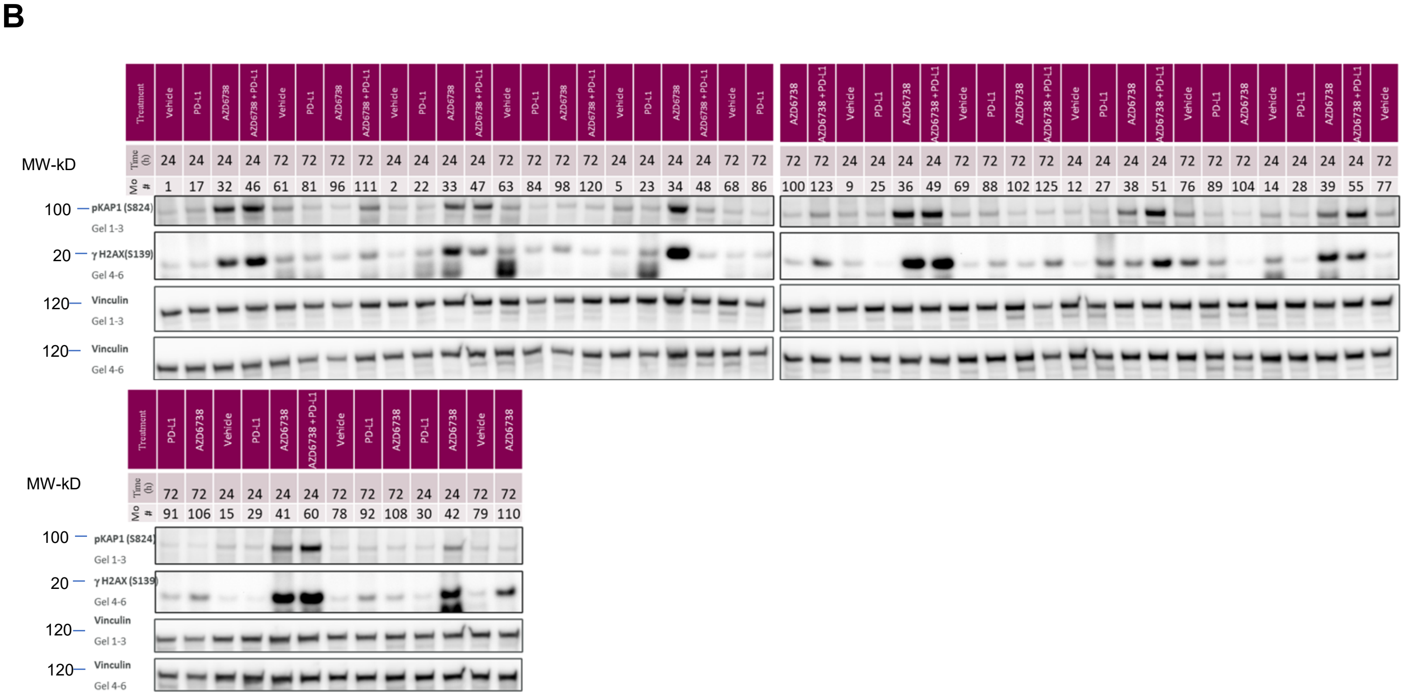

Supplemental Figure 1. ATR target engagement in mouse tumors. A. Western blot quantification of pKAP1 (S824) and γH2AX (S139) induction in CT26 tumors from TB mice dosed at 25 mg/kg b.i.d ceralasertib when on-treatment for 7 days at 2 hours post last dose and then on day 14 when off-treatment (7 days on/7 days off). Data are presented as mean ± SEM percentage change in expression relative to time matched vehicle control, with each dot representing individual tumors. P values calculated by one-way ANOVA. P values >0.05 are not shown. N=7 vehicle treatment group; N=8 all other groups. **B.** Western blot images for quantification data as shown in **A**.

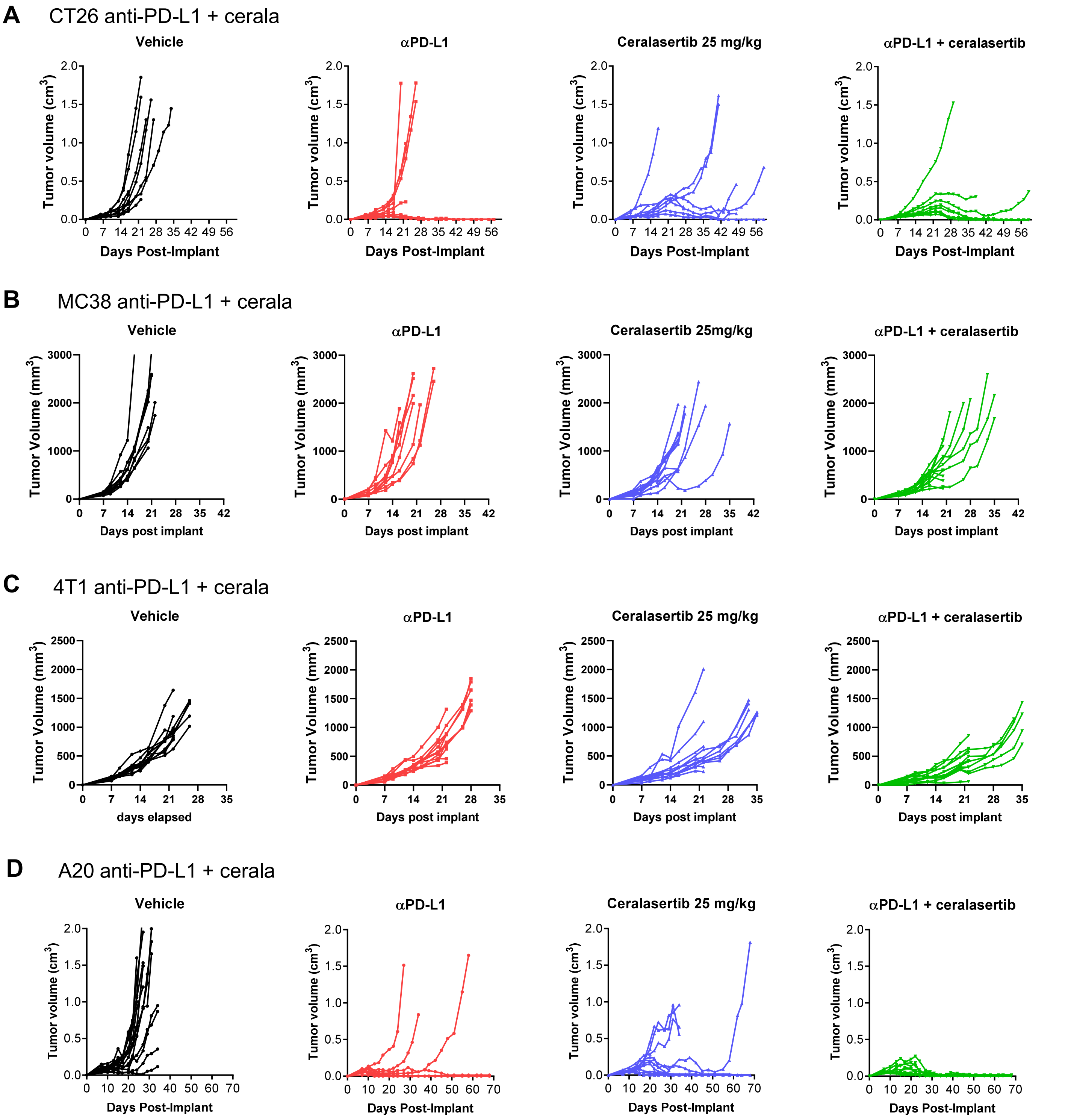

Supplemental Figure 2. Effect of ceralasertib on tumor growth in syngeneic mouse tumor models. Ceralasertib 25 mg/kg BID 7on/7off ± anti-PD-L1 individual tumor volume curves for **A.** CT26 (N = 10), **B.** MC38 (N=9 for vehicle and aPD-L1 groups and N=12 for two other groups), **C.** 4T1 (*n* = 12) and **D.** A20 (N= 10 for aPD-L1 group, N=15 for all other groups) syngeneic mouse tumor models. Individual anima tumor growth curves are shown.

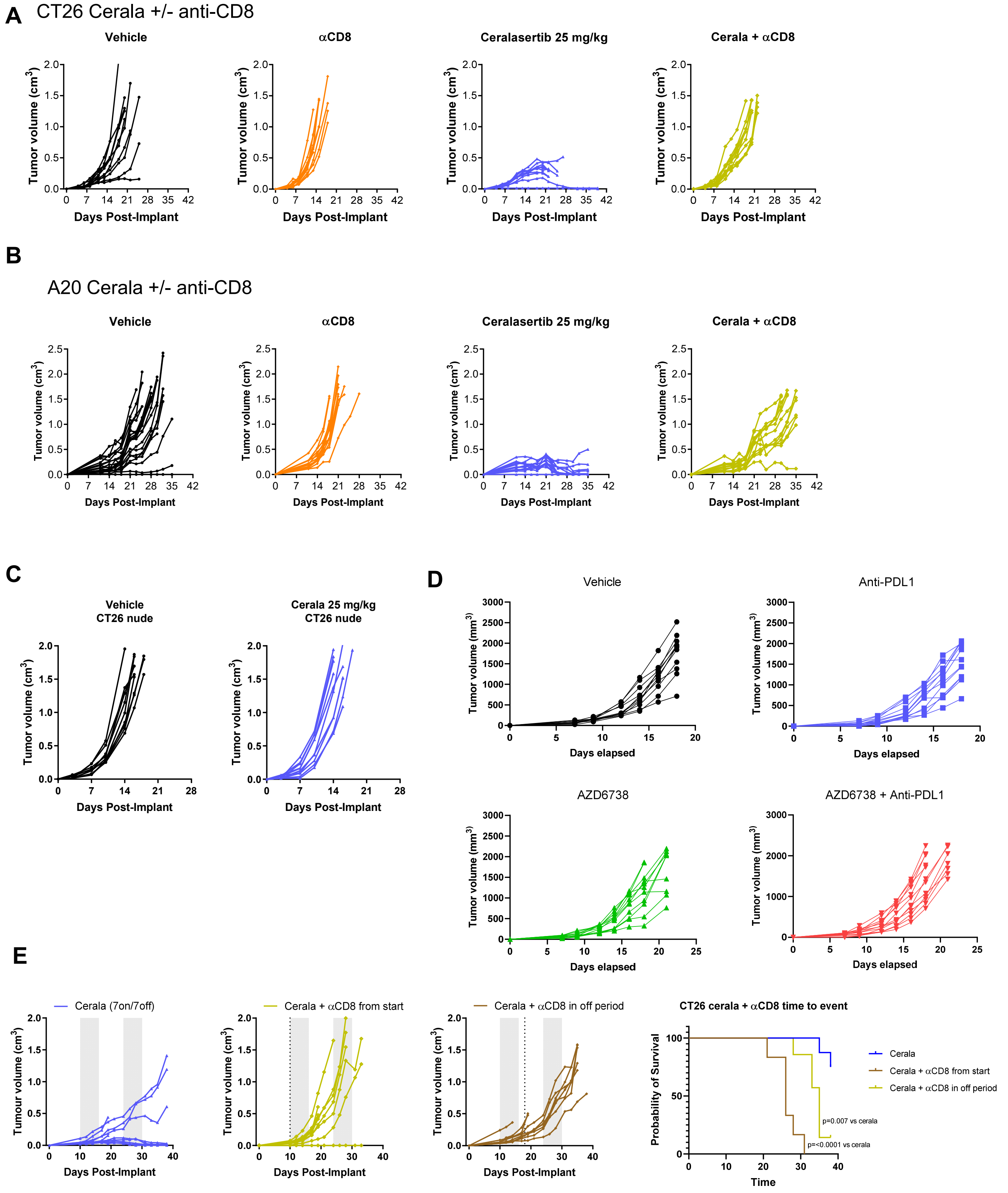

Supplemental Figure 3. Ceralasertib activity is CD8⁺ T-cell dependent. CD8 depletion individual animal tumor volume growth curves for **A.** CT26 (*n* = 10), **B.** A20 (*n* = 20 vehicle group, *n*=12 all other groups) mouse syngeneic tumor models. **C.** Individual animal tumor volume growth curves for ceralasertib treated CT26 tumors in immunodeficient nude mice (*n* = 10). **D.** Nude MC38 TB mice were treated as indicated on graph the same way as in Fig. 1a. Tumor growth in individual mice (n=10) and tumor growth rate are shown. Statistics was calculated in one-way ANOVA with correction for multiple comparisons. ns- not significant. **E.** Individual animal tumor growth curves and survival (time to 1cm^3 tumor volume) following CD8 depleting antibody (αCD8) administered at the start of treatment or in the off-treatment period in CT-26 mouse tumor model (*n* = 10). Ceralasertib daily dosing periods are indicated by grey shaded area and day of start of αCD8 administration indicated by dotted line. P-value Log-rank (Mantel-Cox) test for median survival comparison to ceralasertib monotherapy was used for statistical analysis.

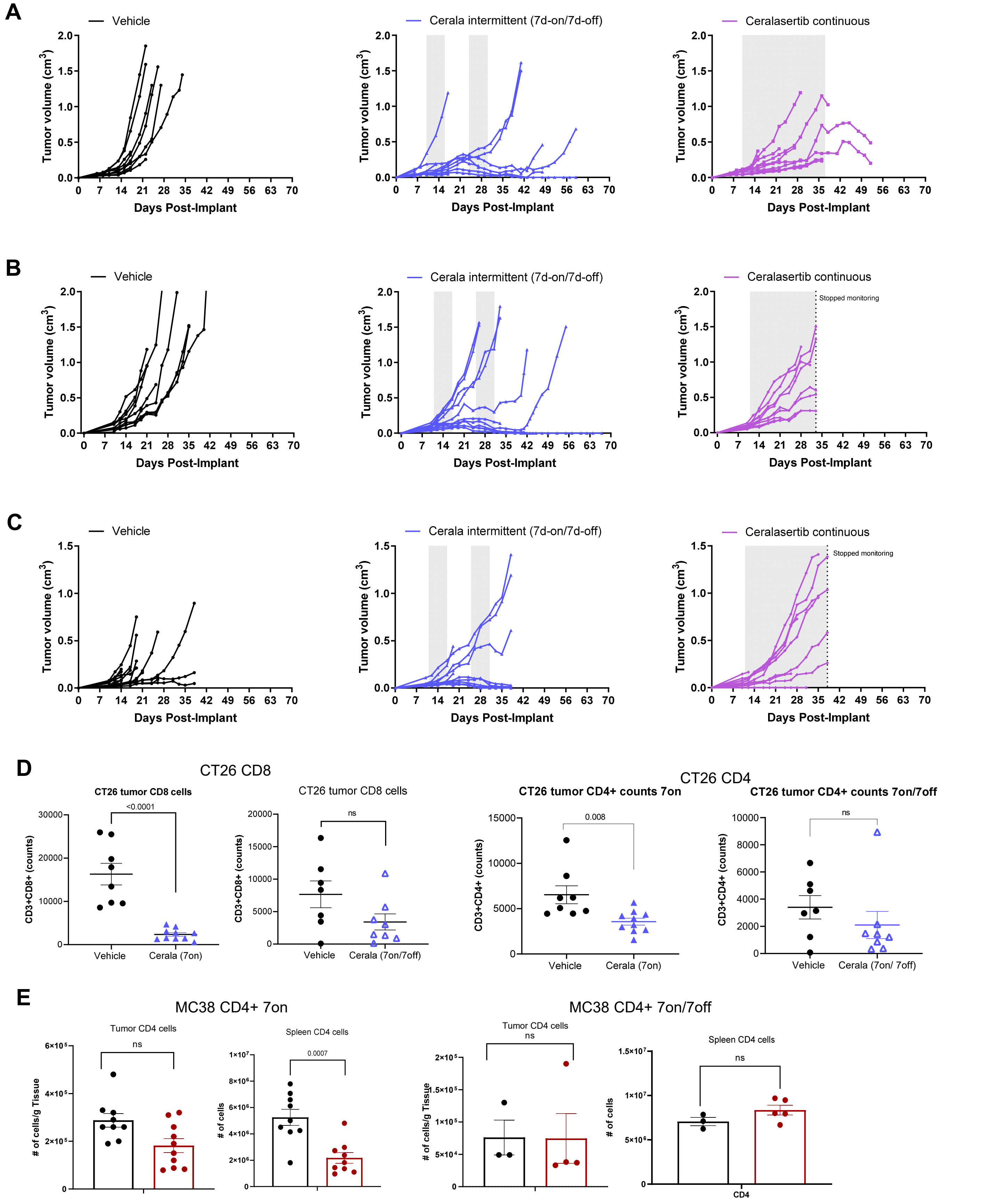

Supplemental Figure 4. Intermittent 7d-on/7d-off schedule improves anti-tumor activity. **A-C.** Individual animal CT26 tumor volume growth curves from 3 independent replicate experiments (N = 10 per experiment). Ceralasertib daily dosing periods with intermittent 7 days-on/7days-off (x2) dosing compared to continuous daily dosing (23-28 days) are indicated by grey shaded areas. Measures stopped for all animals in continuous dosing groups where indicated by a dotted vertical line. **D.** Flow cytometry quantification of CD8+ and CD4+ T-cells in CT26 tumors. N=8 vehicle group, N=10 cerala group. **E.** The number of CD4⁺ T cells in tumor and spleen of MC38 TB mice treated with 25mg/kg ceralasertib either on a 7 days on or 7 days on/7 days off regimen. The number of cells was per gram of tissue. *P<0.05, in one way ANOVA.

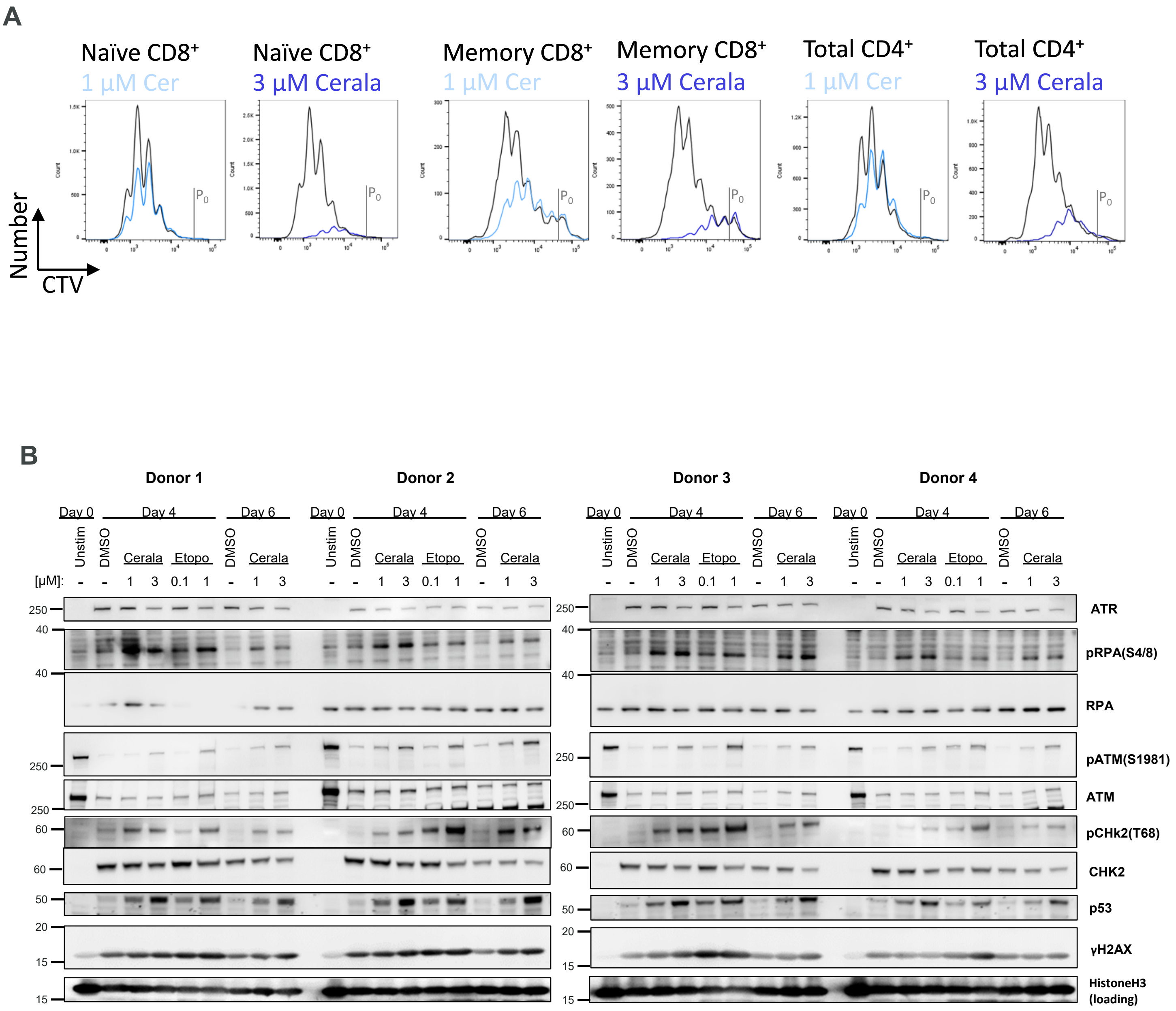

Supplemental Figure 5. Analysis of human T-cells isolated from healthy human donor volunteer PBMCs (N = 4) and stimulated to proliferate *ex vivo* with CD3/CD28 beads in the absence of presence of ceralasertib. A. Representative flow cytometry CellTrace Violet (CTV) cycle plots for stimulated CD3+ T-cells treated with 1 or 2 µM ceralasertib. **B.** Western blot analysis of ATM-ATR signalling and DNA damage induction in unstimulated (Day 0) and stimulated CD3+ T-cells in presence of vehicle (DMSO), 1 or 3 uM ceralasertib or 1 uM etoposide +ve control for 4 or 6 days from 4 different human volunteer donors.

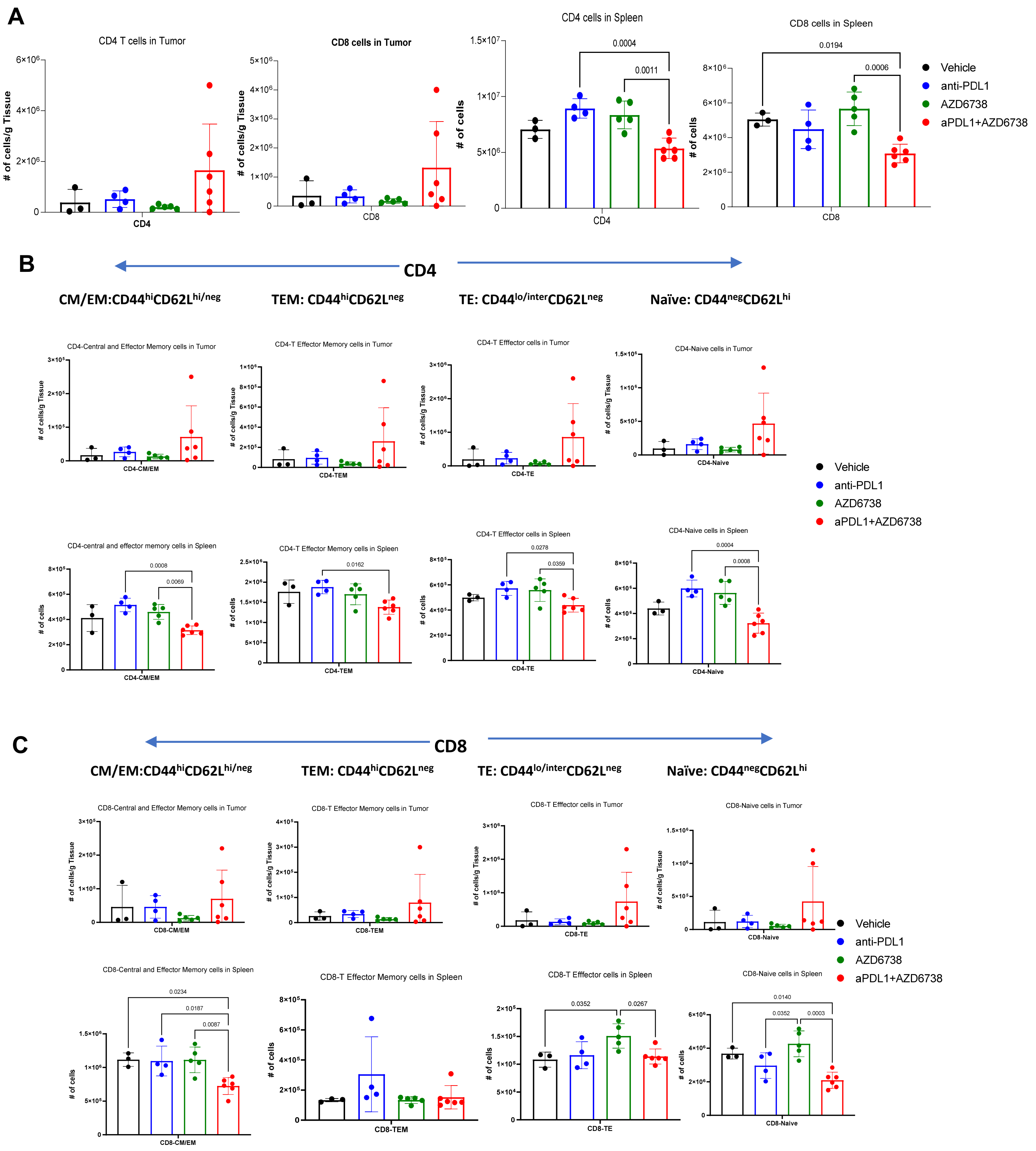

Supplemental Figure 6. Impact of ceralasertib in combination with anti-PD-L1 of T cell subsets. The number of CD4+ and CD8+ T cells tumors and spleens of MC38 TB mice treated with 25 mg/kg of ceralasertib 7 days on or 7 days on/7 days off in combination with 10 mg/kg of anti-PDL1 (clone 10F.9G2). The treatment was initiated 5 days post implantation. **A.** The number CD4+ and CD8+ T cells; **B,C.** The number of indicated T cell subsets: T Effector (TE): $CD44^{lo/inter}CD62L^{neg}$; T Central/Effector Memory (CM/EM): $CD44^{hi}CD62L^{hi/neg}$; T Effector memory (EM):$CD44^{hi}CD62L^{neg}$; T Naïve: $CD44^{neg}CD62L^{hi}$; The number of cells for each subset was calculated as the number of cells per gram of tissue. Results of individual mice are shown. P values were calculated in one-way ANOVA. Only p values < 0.05 are shown. N=3 vehicle group; N= 4 anti-PD-L1 group; N = 5 AZD6738 group; N= 6 combination group.

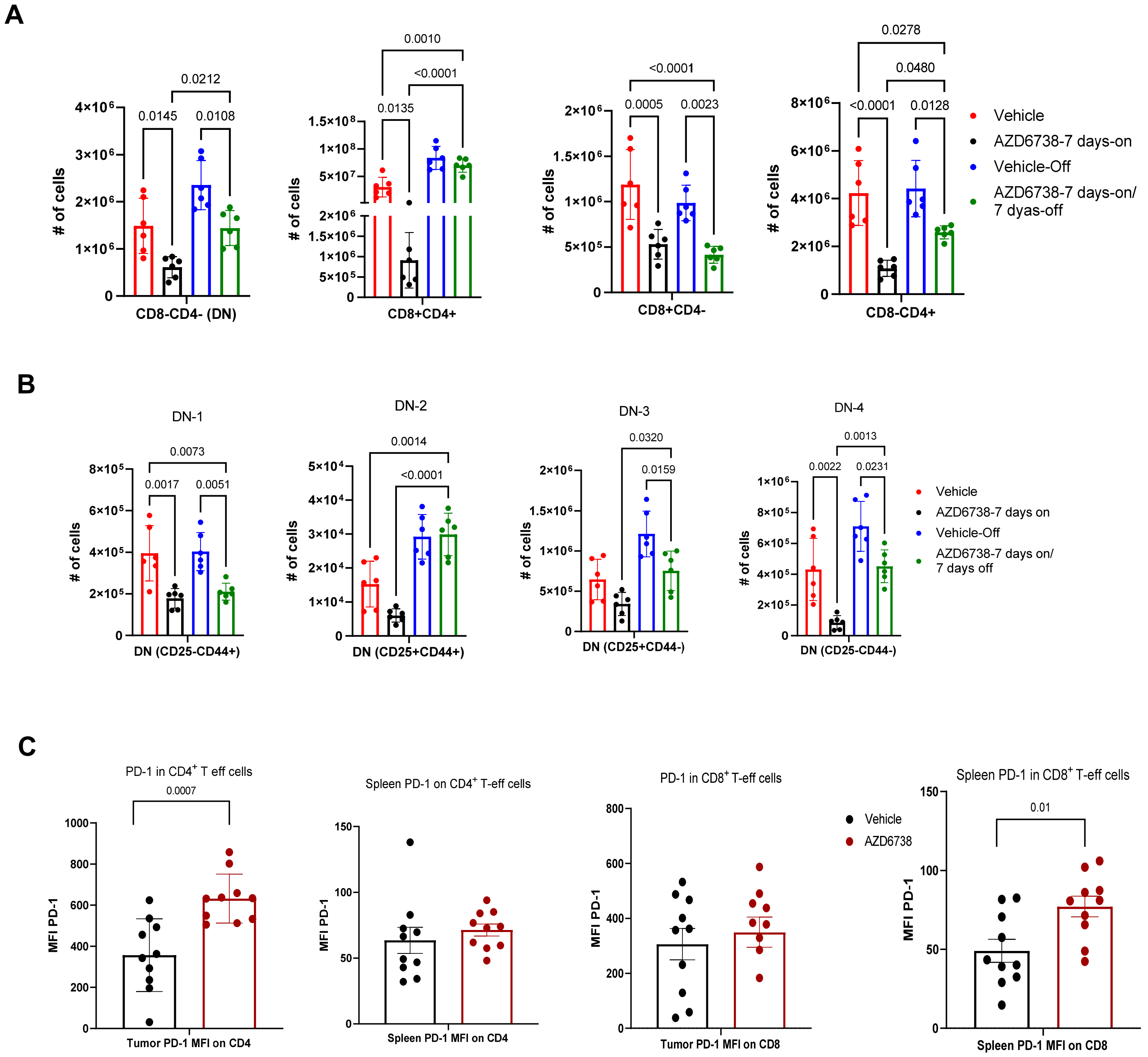

Supplemental Figure 7. Effect of ceralasertib on thymic T cell precursors. MC38 tumor-bearing mice were treated for 7 days with 25 mg bid (7-days on) and then left untreated for 7 days (7 days on/ 7 days off). Thymocytes were evaluated as shown on graph. **A.** The populations of thymocytes. **B.** Analysis of double negative (DN) $CD4^-CD8^-$ precursors. The number of cells were calculated per thymus. **C.** The Mean Fluorescence Intensity (MFI) of PD1 expression on CD4+ and CD8+ T cells. Individual data, mean and SD are shown; P values were calculated in One-way ANOVA with correction for multiple comparisons. N=5 representing individual mice. Only p values < 0.05 are shown.

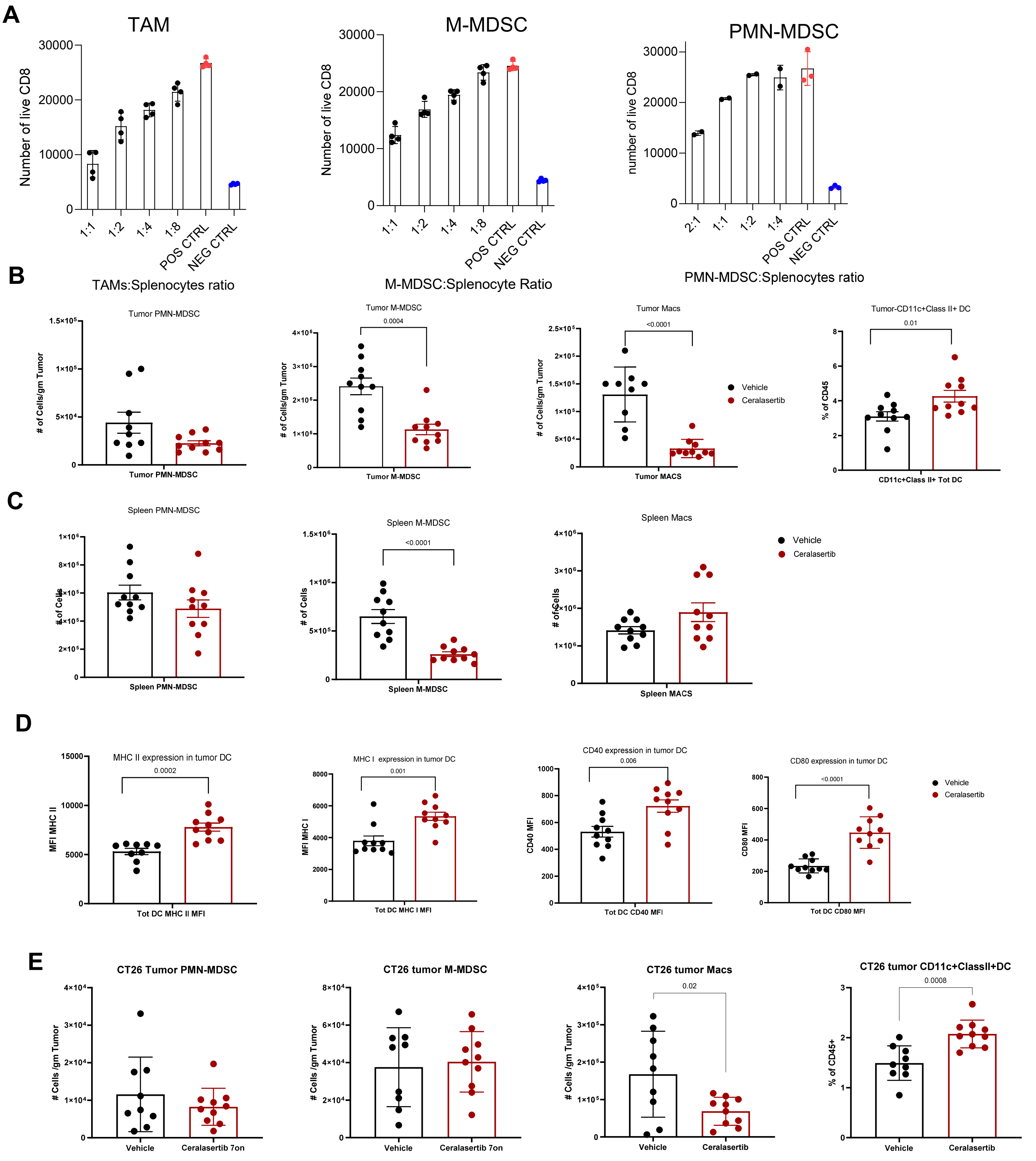

Supplemental Figure 8. Impact of ceralasertib on myeloid cells in tumor and spleen. A. Tumor infiltrating TAMs, M-MDSC and PMN-MDSC were isolated from MC38 TB mice and assessed for their ability to suppress T cells proliferation in a standard suppression assay using PMEL and gp100 peptide to stimulate T cells. 50,000 PMEL were plated per each well with different ratio of myeloid cells and $CD8^+$ T cells. The number of T cells was counted by flow cytometry. n=4 per group representing individual mice. **B-D**.The number of PMN-MDSC, MDSC, macrophages (Macs) and dendritic cells (DC) tumors and spleens. MC38 tumor bearing mice were treated 14 days post implantation with 25 mg/kg b.i.d. of ceralasertib for 7 days and cell number or MFI was determined by flow cytometry. N=9 vehicle group; N= 10 ceralasertib group. **B-C.** the number of myeloid cell subsets in tumors; PMN-MDSC: $CD11b^+Ly6G^{hi}Ly6C^{lo/neg}$, M-MDSC:$CD11b^+Ly6G^{lo/neg}Ly6C^{hi}$, Macs: $F4/80^+CD11b^+LyG^-Ly6C^-$, DC:$CD11c^+$MHC II^+ Tumor. **D.** the MFI of DC activation markers. . **E.** Indicated myeloid cells in tumors of CT26 TB mice treated with 50 mg/kg q.d. of ceralasertib for 7 days. Cell number was determined by flow cytometry. Phenotype was determined as described for MC38 TB mice. N=9 vehicle group; N= 10 ceralasertib group. P values were calculated in one way ANOVA. Results of individual mice, mean and SD are shown. Only p values < 0.05 are shown.

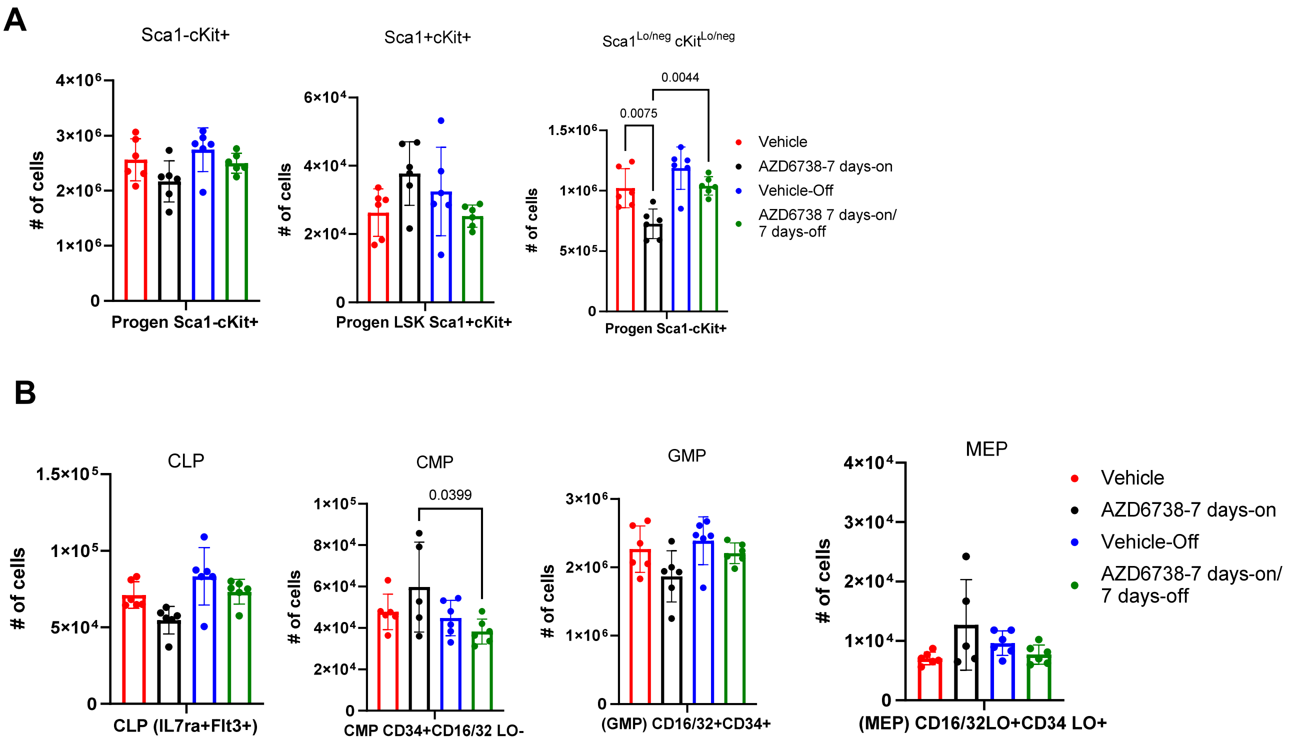

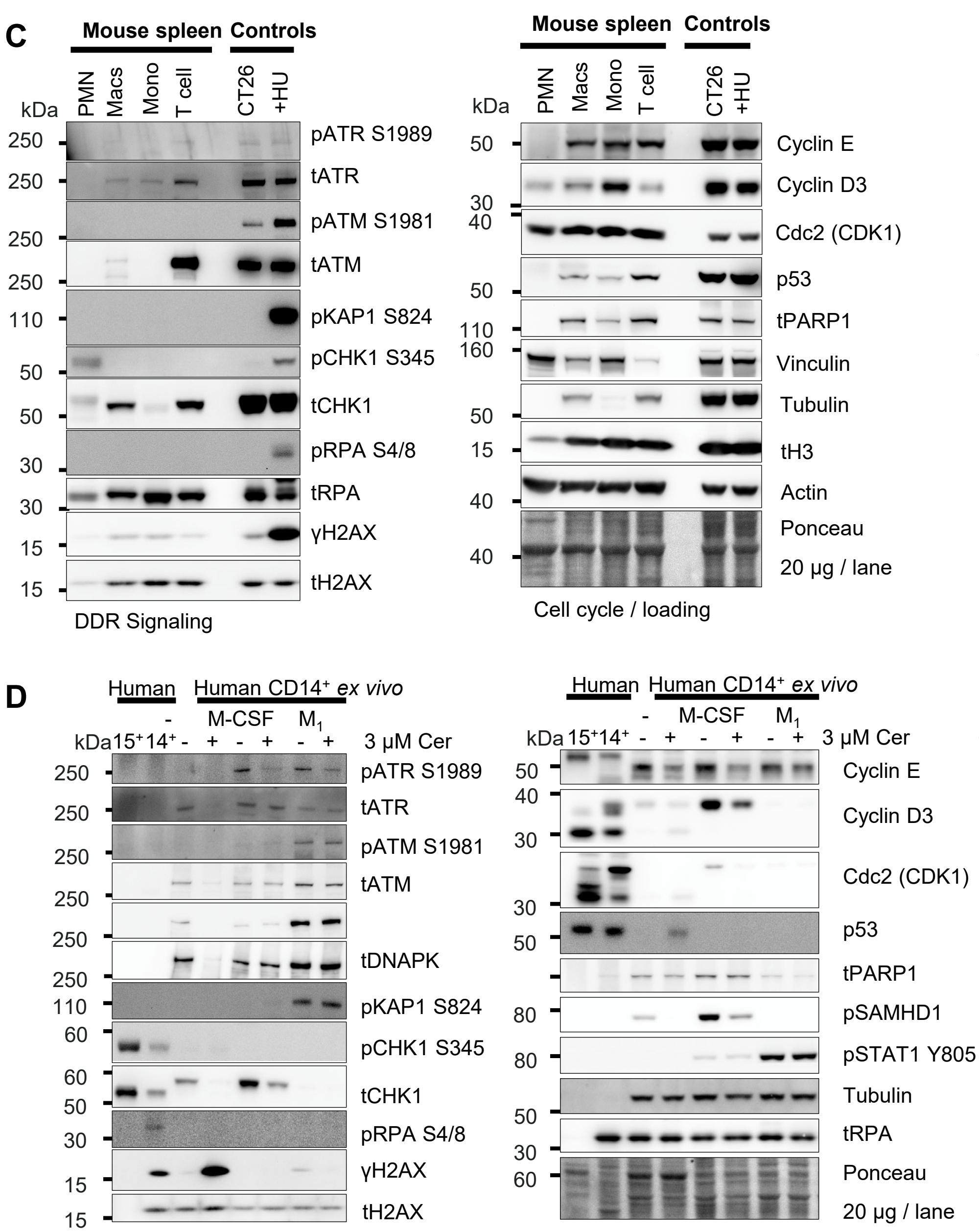

Supplemental Figure 9. Effect of ceralasertib treatment on bone marrow myeloid progenitors. MC38 tumor-bearing mice were treated and evaluated as described in Fig.1a. **A.** Populations of early myeloid progenitors. **B.** Populations of late myeloid progenitors. Individual data, mean and SD are shown. P values were calculated in One-way ANOVA with correction for multiple comparisons. N=5 representing individual mice. Only p values < 0.05 are shown. **C, D.** Components of DDR in different cells. **C.** Indicated populations of splenocytes were sorted from MC38 TB mice, lysed, and immunoblotted for indicated DDR and cell cycle markers. Mouse CT-26 tumor cells were used to generate positive control lysates for DDR signaling using 2 mM HU. Two experiments with the same results were performed. **D**. Human CD15+ PMN and CD14+ monocytes were isolated from healthy donors and either lysed immediately or cultured *ex vivo* for 6 to 8 days as indicated, before lysis and immunblotting for the indicated DDR and cell cycle markers. Two experiments with the same results were performed.

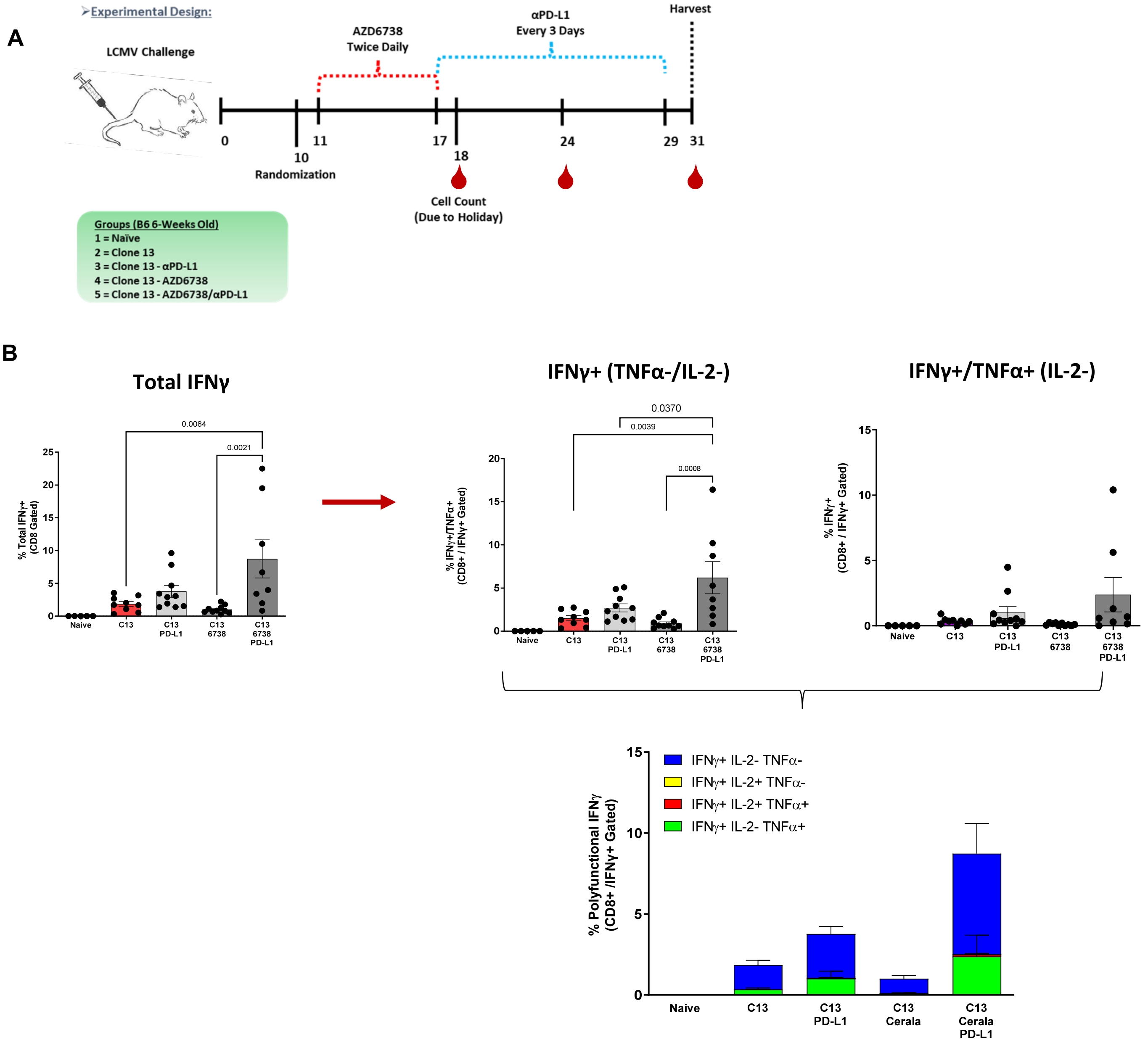

Supplemental Figure 10. Effect of ceralasertib on immune response in the LCMV mouse model. A. Schematical representation of the LCMV Clone 13 experimental design including a combination dosing of Ceralasertib and anti-PD-L1. **B.** Proportion of IFNγ and TNFα positive gp33^{+} CD8^{+} T cells in spleens after restimulation with gp33-derived peptide measured by flow cytometry. N=8 representing individual mice. P values were calculated in one-way ANOVA. Only p values < 0.05 are shown.

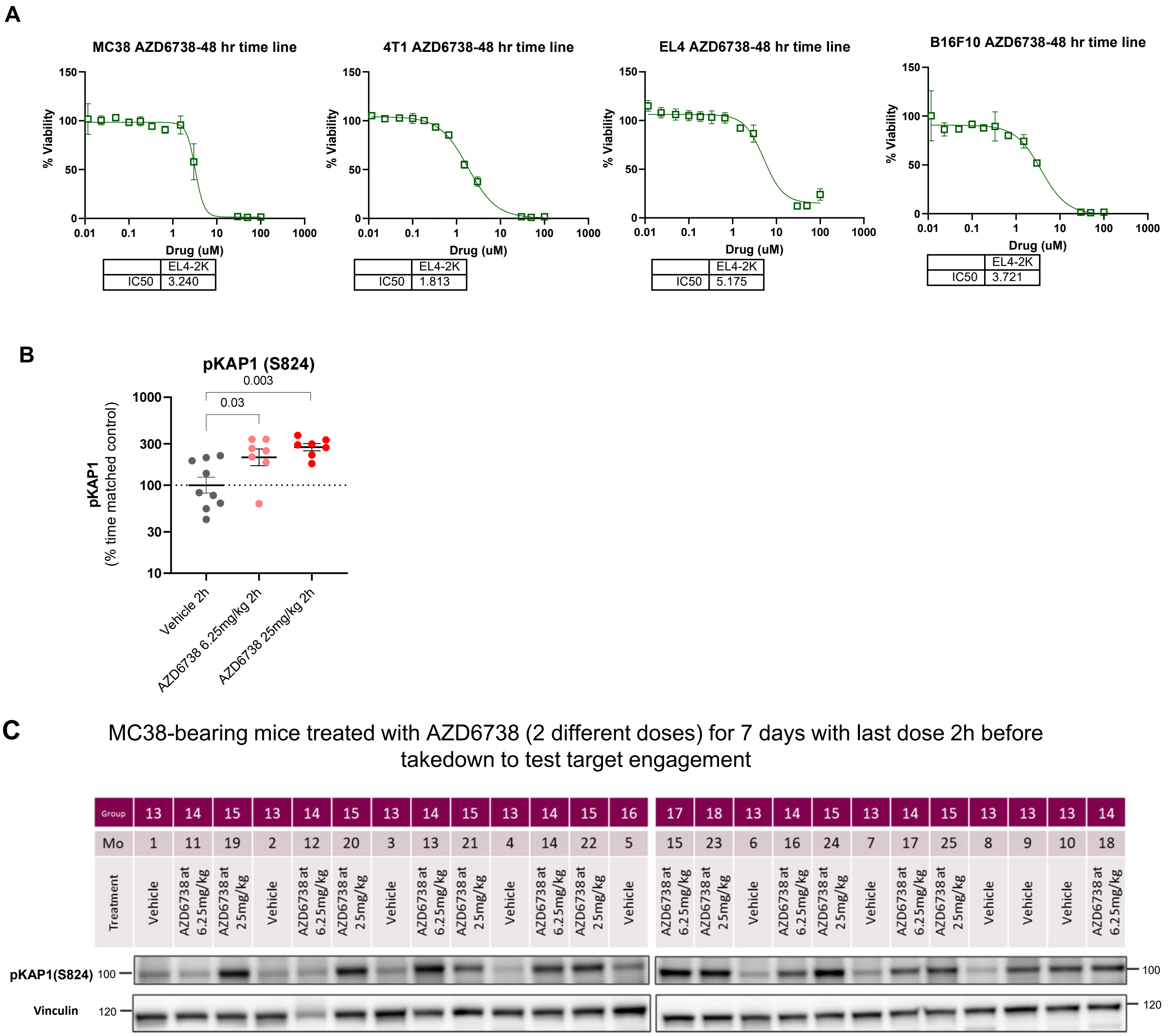

Supplemental Figure 11. Low dose AZD6738 does not impair tumor cells viability. A. Cyotoxicity of ceralasertib for 4 murine tumor lines was determined *in vitro* to identify dose appropriate to evaluate mechanism regulating sensitization of tumors to check-point inhibitor. Tumor cells were grown to confluency, harvested and seeded in 384-well plate at density to facilitate growth during treatment. Tumor cells were treated with titrating dose of AZD6738 for 48 hours and cell viability measured using the CellTiter-Glo™ Luminescent Cell Viability Assay according to manufacturer protocol. IC50 were calculated from a x-y plot using best fit curve with GraphPad Prism. aPD-L1 + ceralasertib 25 mg/kg versus 6.25 mg/kg anti-tumor growth inhibition in CT26 model. **B.** Western blot protein quantification of pKAP1 (S824) DNA damage marker induction in MC38 tumors from TB mice when on-treatment with low dose 6.25 mg/kg b.i.d. or standard dose 25 mg/kg b.i.d.for 7 days at 2 hours post last dose. Data are presented as mean ± SEM percentage change in expression relative to time matched vehicle control, with each dot representing individual tumors. N=7 cerala group; N=9 vehicle treated group. P values were calculated in one-way ANOVA with correction for multiple comparisons. **C.** Western blot images used for quantification data as shown in **B**.

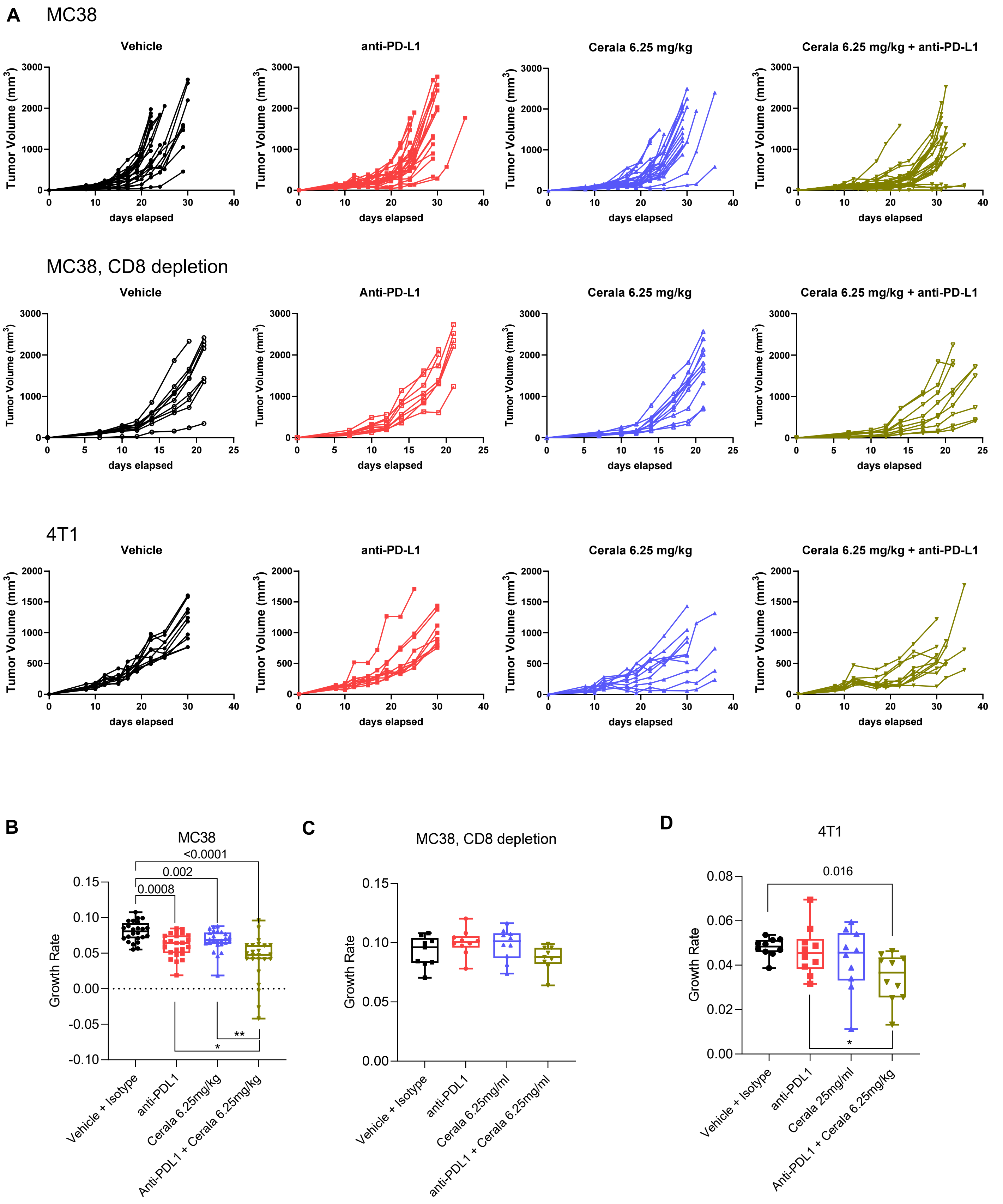

Supplemental Figure 12 Antitumor effect of combination of low dose ceralasertib with anti-PD-L1. A. Tumor growth curves of MC38 and 4T1 TB mice treated with ceralasertib at 6.25mg/kg starting at day 3 with a 7 days on/ 7 off schedule in combination with anti-PDL1 (10mg/kg twice a week). Where indicated mice were treated with CD8 depleting antibody. **B-D.** Growth rate of MC38 and 4T1 treated with Cerlasertib 6.25mg/kg with or without anti-PDL1. Where indicated the experiment was performed in CD8-depleted mice. Each dot represent a single mouse. N=10 per group. P values were calculated in one-way ANOVA with correction for multiple comparisons. Only p values <0.05 are shown.

A

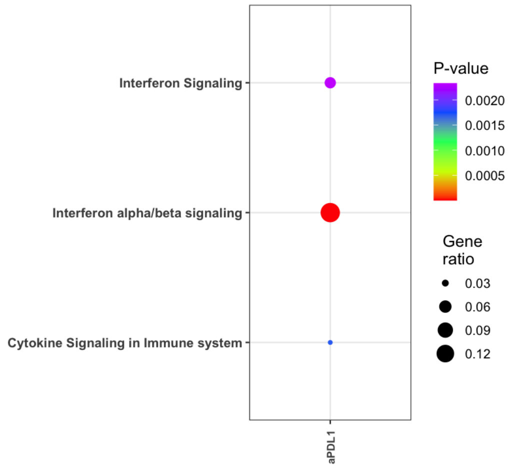

B

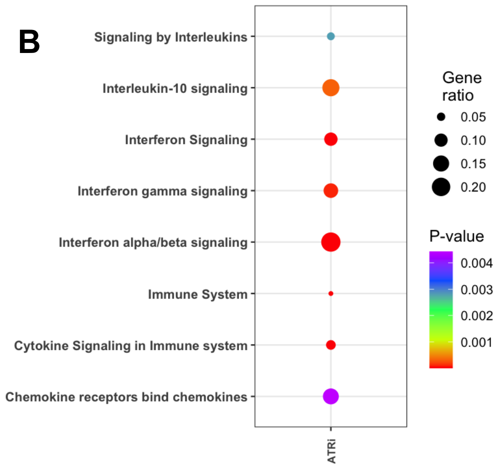

C

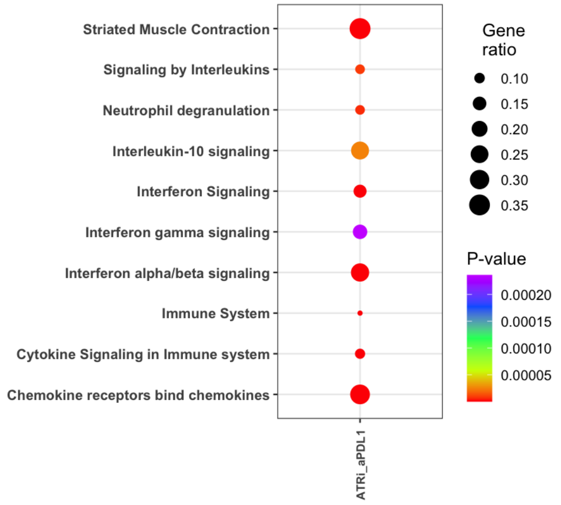

d

Term name	Comparison vs vehicle	Overlap size (# genes)	Term size total (# genes)	Ratio (overlap/total)	P-value (vs vehicle)
Chemokine receptors bind chemokines	Ceralasertib (UP)	7	48	0.15	0.00443
Chemokine receptors bind chemokines	Combo (UP)	15	48	0.31	2.20E-08
Cytokine Signaling in Immune system	Ceralasertib (UP)	41	687	0.06	9.82E-11
Cytokine Signaling in Immune system	Combo (UP)	66	687	0.1	2.30E-11
Immune System	Ceralasertib (UP)	79	2019	0.04	7.30E-13
Immune System	Combo (UP)	136	2019	0.07	7.56E-13
Interferon alpha/beta signaling	αPD-L1 (UP)	10	68	0.15	9.51E-08
Interferon alpha/beta signaling	Ceralasertib (UP)	15	68	0.22	7.64E-11
Interferon alpha/beta signaling	Combo (UP)	18	68	0.26	5.81E-09
Interferon Signaling	αPD-L1 (UP)	10	195	0.05	0.00233
Interferon Signaling	Ceralasertib (UP)	20	195	0.1	2.19E-08
Interferon Signaling	Combo (UP)	27	195	0.14	4.99E-07
Interleukin-10 signaling	Ceralasertib (UP)	8	47	0.17	0.000308
Interleukin-10 signaling	Combo (UP)	12	47	0.26	2.46E-05
Interferon gamma signaling	Ceralasertib (UP)	11	90	0.12	8.82E-05
Interferon gamma signaling	Combo (UP)	15	90	0.17	0.000236
Signaling by Interleukins	Ceralasertib (UP)	22	464	0.05	0.00281
Neutrophil degranulation	Combo (UP)	43	476	0.09	5.95E-06
Signaling by Interleukins	Combo (UP)	42	464	0.09	8.40E-06
Striated Muscle Contraction	Combo (UP)	12	34	0.35	3.92E-07

Supplemental Figure 13. Unbiased bulk RNAseq REACTOME pathways gene enrichment analysis in CT26 tumor model. Tumors were collected at the end of the 7 days on/7 days off ceralasertib treatment cycle. **A.** Anti-PD-L1 vs vehicle. N=8 in anti-PD-L1 group, N=8 in vehicle group. **B.** Ceralasertib vs Vehicle. N=11 in ceralasertib group **C.** Ceralasertib + anti-PD-L1 combination vs Vehicle. N=9 combination group. **D.** Table of significantly enriched gene pathway terms. Only up-regulation of pathways were found to be significantly enriched upon treatment.

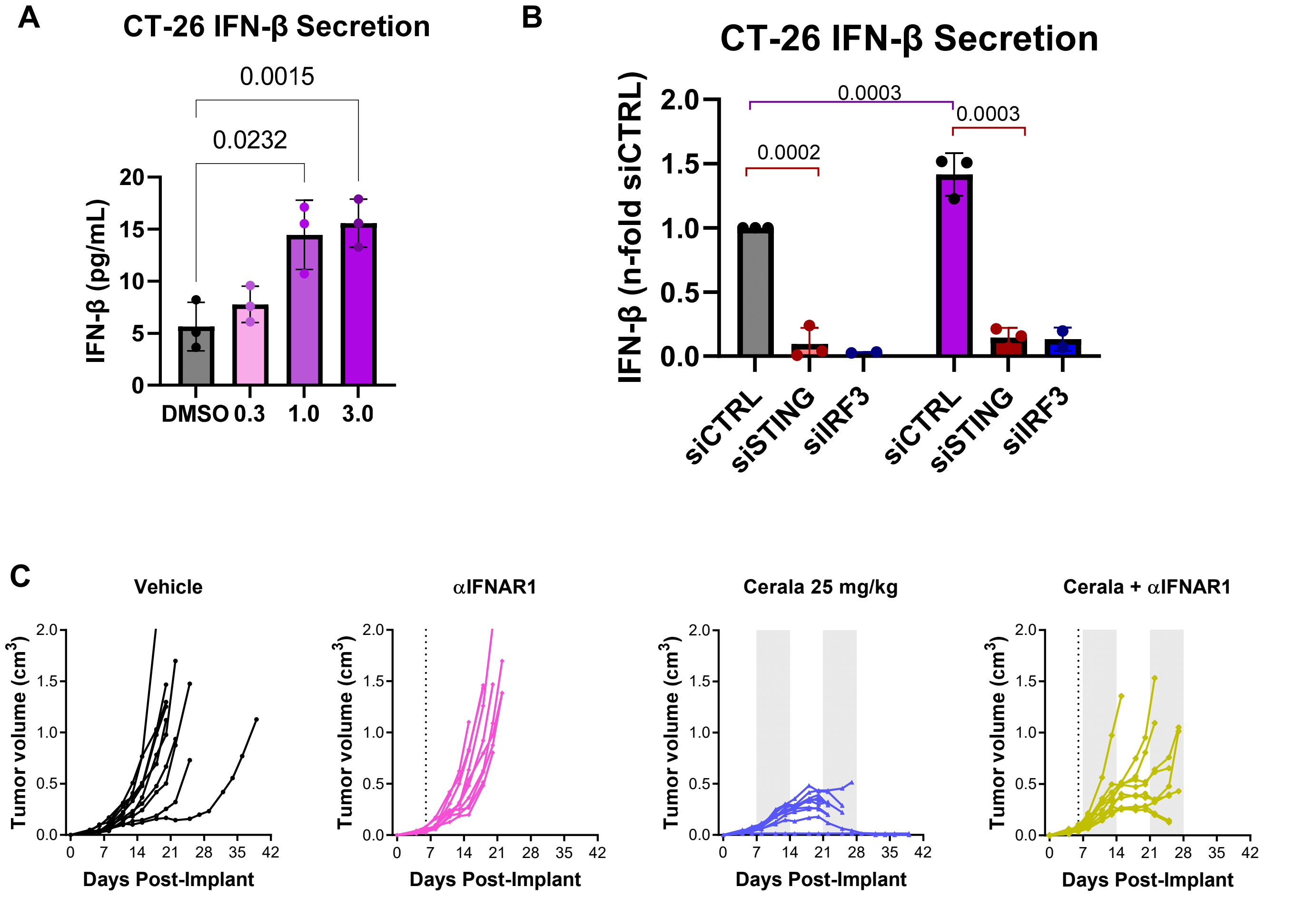

Supplemental Figure 14. Ceralasertib STING and IFN dependent responses in CT26 mouse tumor model. A. Induction of IFN-β secretion from CT26 tumor cells cultures treated with indicated concentrations of ceralasertib *in vitro* for 72 hours. Data are from one experiment with 3 technical replicates is shown. P values were calculated with One-way ANOVA with correction for multiple comparisons. P>0.05 are not shown. **B.** Quantification of IFN-β secretion from CT26 tumor cells cultures treated with ceralasertib and siRNA to STING (siSTING) or IRF3 (siIRF3). Statistics indicate >99.9% confidence. Two-sided unpaired Student's t-test was used. **C.** Individual *in vivo* tumor growth curves of CT26 TB mice treated (n = 10) with ceralasertib (7d-on/7d-off), INAFR1 blocking antibody (αIFNAR1) of the combination. Ceralasertib daily dosing periods are indicated by grey shaded areas and start of αIFNAR1 administration indicated by dotted line.

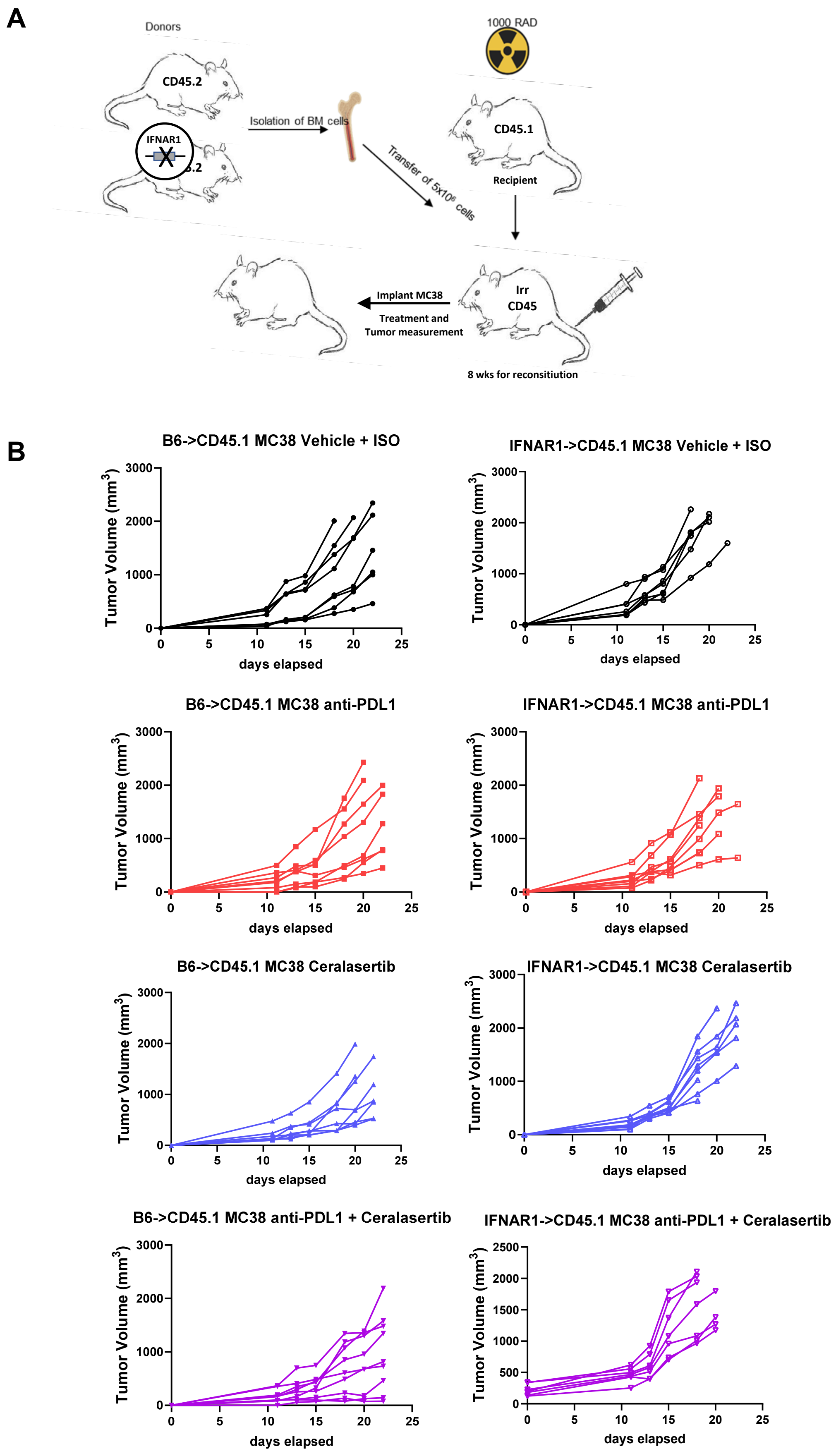

Supplemental Figure 15. Tumor growth in mice reconstituted with bone marrow from IFNAR KO mice. A. Schematic representation of IFNAR1 KO bone marrow chimera generation and tumor implantation. **B.** Individual growth curves of MC38 tumors in mice reconstituted with BM from WT (on the left) or IFNAR1 KO (on the right) treated with ceralasertib (AZD6738) 6.25mg/kg and anti-PDL1. ISO – control IgG. N=8 except for N=9 B6->CD45.1 WT MC38 anti-PDL1+ Ceralasertib; N=7 IFNAR1->CD45.1 MC38 Vehicle and anti-PDL1 + ceralasertib.

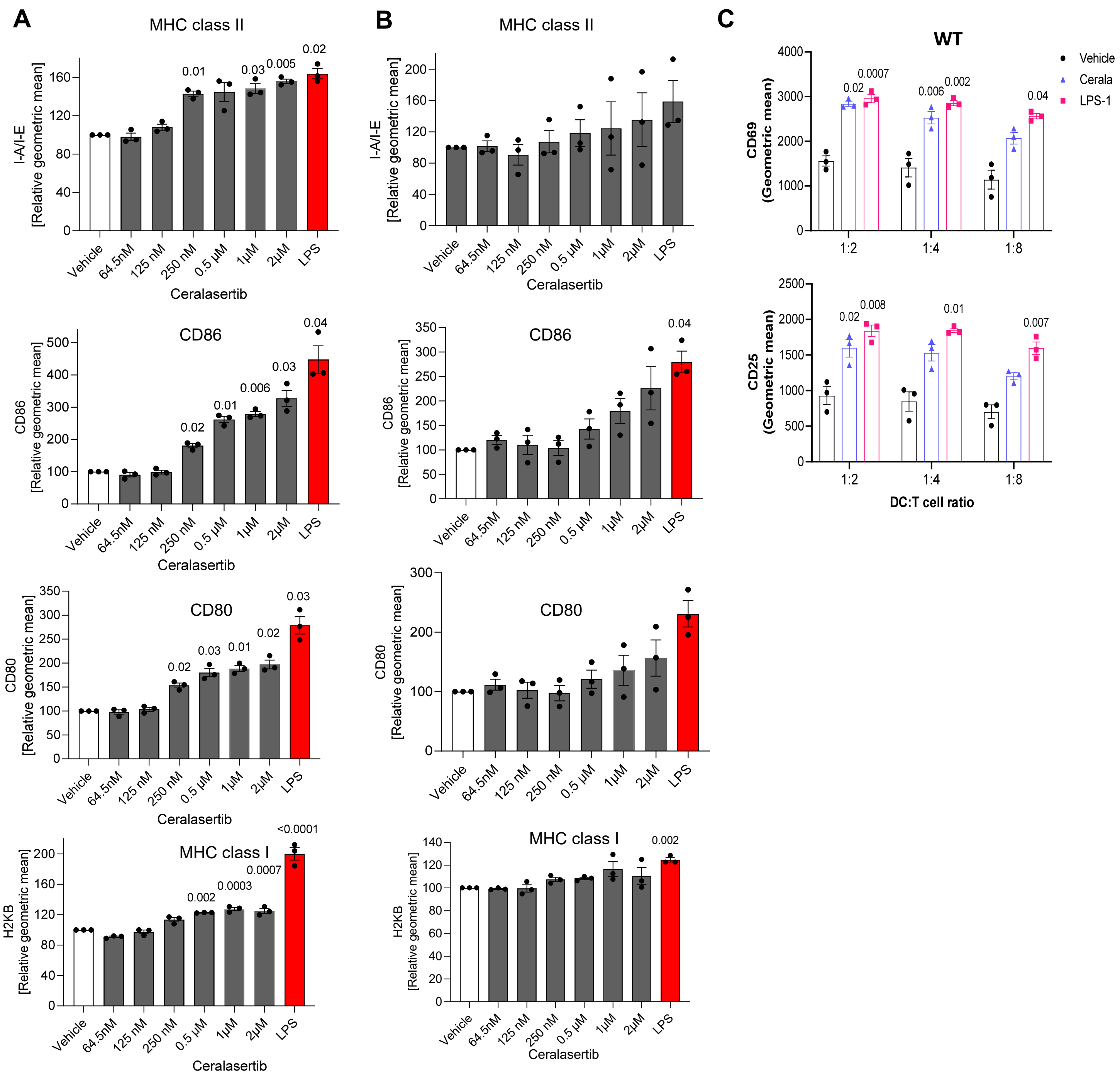

Supplemental Figure 16. Effect of ceralasertib on DC activation. DCs were generated from BM with GM-CSF and IL-4. Cells were treated with different concentrations of ceralasertib (AZD6738) or 10ng/ml LPS. (positive control) **A.** Mean fluorescent intensity of expression of indicated molecules in cells generated from WT mice. N=3. **B.** Mean fluorescent intensity of expression of indicated molecules in cells generated from IFNAR KO mice. N=3. **C.** Activation of T cells by DCs treated with ceralasertib and LPS (as a positive control). CD25 and CD69 were used as makers of T-cell activation. N=3. P values were calculated in one-way ANOVA test with correction for multiple comparisons. Only p values <0.05 are shown.

A

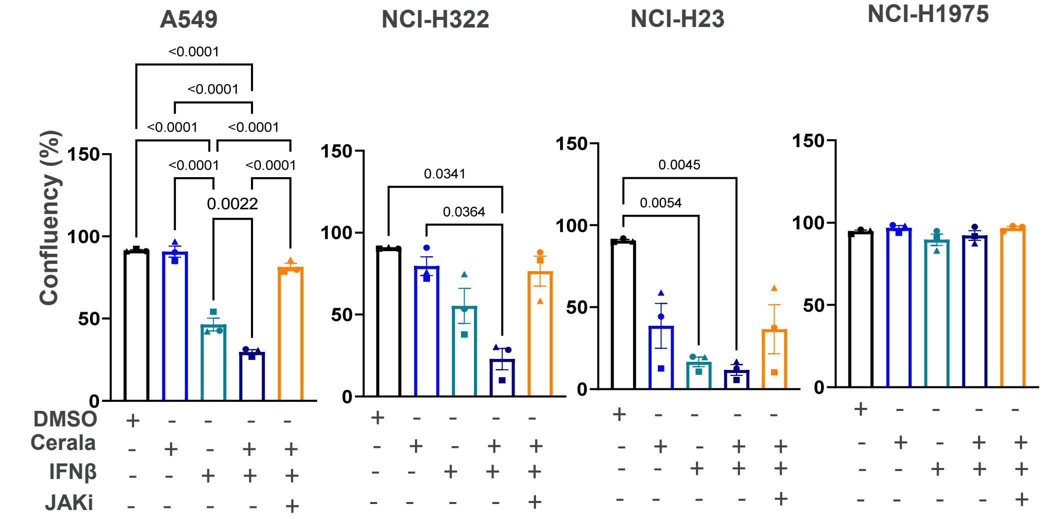

B

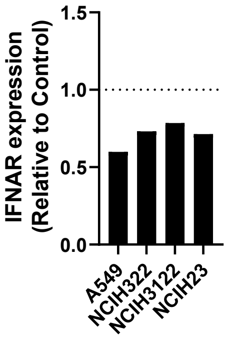

C

	p-value versus DMSO vehicle control			p-value versus ATRi + IFNβ	
Cell Line	ATRi	IFN-β	ATRi + IFN-β	ATRi	IFN-β
NCIH23	0.019066 *	0.000017 ****	0.000017 ****	NS	NS
NCIH1573	0.001847 **	0.000464 ***	0.000464 ***	NS	0.035856 *
NCIH3122	NS	0.005076 *	0.005076 *	0.004827 **	NS
NCIH1993	NS	0.031659 *	0.031659 *	0.032741 *	NS
NCIH838	NS	0.000037 ****	0.000037 ****	0.000053 ****	0.016273 *
NCIH322	NS	0.029948 *	0.029948 *	0.00278 **	NS
A549	NS	0.000342 ***	0.000342 ***	0.000076 ****	0.01612 *
SW1573	NS	0.012944 *	0.012944 *	NS	NS
NCIH1299	NS	0.000332 ***	0.000332 ***	0.003939 **	NS
NCIH460	NS	NS	NS	0.054542 *	NS
NCIH2009	0.026795 *	NS	NS	0.027384 *	0.019196 *
PC9	0.044752 *	NS	NS	NS	0.012045 *
NCIH441	NS	NS	NS	NS	NS
NCH1975	NS	NS	NS	NS	NS
NCIH1650	NS	NS	NS	NS	NS
NCIH2085	NS	NS	NS	NS	NS
NCI-H1437	-	0.039088 *	-	-	-

Statistics (p-values) were calculated by student's t-test (GrpahPad Prism). NS non-signicant p > 0.05, * p ≤ 0.05, ** p ≤ 0.01, *** p ≤ 0.001, **** p ≤ 0.0001.

Suppemental Figure 17. Ceralasertib plus IFN-β signalling enhances growth inhibition of tumor cell lines *in vitro.* A. growth inhibitory activity of monotherapy ceralasertib (1 µM), IFN-β (1 ng/ml), combination of ceralasertib and IFN-β and ceralasertib and IFN-β with the JAK inhibitor (5 µM) in 4 representative human lung cancer cell lines. Each condition was normalised to the DMSO control and the mean ± S.E.M for three biological replicates for each cell line is represented as a bar graph. Statistical significance by one-way ANOVA test with correction for multiple comparisons is indicated on the graphs. **B**. TaqMan qRT-PCR relative RNA expression quantification of IFNAR1 siRNA mediated knockdown in human lung cancer cell lines. **C.** Table of statistical comparisons for each treatment group as indicated in 16 NSCLC cell line panel.

Supplemental Table 1. Antibodies used for flow cytometry. All antibodies were from BD bioscience

Name of antibody	Clone	Dilutions
CD16/32	2.4G2	100
CD48	HM48-1	200
CD34	RAM34	50
Ly6G	1A8	100
Ly6C	HK1.4	400
CX3CR1	SA011F11	100
CD150 (SLAM)	TC15-12F12.2	100
CD117 (ckit)	3C11	100
sca1 (Ly6A/E)	E13-161.7	100
CD135 (Flt3)	A2F10	50
CD41	MWReg30	200
CD115	AFS98	50
CD127 (IL-7Ra)	A7R34	100
CD19	1D3	100
B220	RA3-6B2	100
CD3	17A2	100
TCRb	H57-597	100
NK1.1	S17016D	100
Ter119	TER-119	100
CD11b	M1/70	100
CD45	30-F11	200
CD19	1D3	200
CD3	17A2	100
CD4	RM4-5	100
CD8a	53-6.7	200
TCRb	H57-597	100
CD24	M1/69	200
CD44	S7 (got IM7)	200
yd TCR	GL3	100
CD25	PC61	100
CD69	H1.2F3	100
CD117 (ckit)	2B8	100
sca1 (Ly6A/E)	E13-161.7	100

Supplemental Table 2. CyTOF antibody panel.

Tag	Target	Clone	Supplier	Order code	Antibody dilution
089Y	CD45	30-F11	Standard biotools	3089005B	1000
111Cd	Ly6G	1A8	Biolegend	127637	2000
112Cd	Ly6C	HK1.4	Biolegend	128039	3000
113Cd	iNOS	CXNFT	Thermo Fisher Scientific	14-5920-82	200
114Cd	CD31	390	Biolegend	102425	1000
115In	Ki-67	B56	Abcam	ab279653	100
116 Cd	CD206	MMR	Biolegend	141702	1000
141Pr	OX40 (CD134)	OX86	Biolegend	119429 l	100
142Nd	CXCR5	LT38D7	Standard biotools	3142015C	120
143Nd	GITR/CD357	DTA1	Standard biotools	3143019B	1000
144Nd	H2-Db/ MHC class I	28-14-8	Standard biotools	3144016b	1000
145Nd	CD4	RM4-5	Standard biotools	3145002B	100
146Nd	F4/80	BM8	Standard biotools	3146008B	500
147S	FasL (CD95L)	Polyclonal	R&D systems	AF526	120
148Nd	CD11b	M1/70	Standard biotools	3148003B	5000
149Sm	Klrg1	2F1	Thermo Fisher Scientific	16-5893-82	1000
150Nd	CD25	3C7	Standard biotools	3150002C	100
151Eu	TCF1	C63/D9	CST	CST2203BF	100
152Sm	CD3e	145-2C11	Standard biotools	3152004b	1000
153Eu	PD-L1 /CD274	10F.9G2	Standard biotools	3153016b	2000
154Sm	CTLA4/CD152	UC10-4B9	Standard biotools	3154008b	100
155Gd	4-1BB	Polyclonal	R&D systems	AF937	500
156Gd	anti-PE (anti-IFNg-PE)	PE001	Standard biotools	3156005C	100
158Gd	Foxp3	FJK-16s	Standard biotools	3158003a	120
159Tb	PD1 /CD279	29F.1A12	Standard biotools	3159024B	120
160Gd	anti-FITC (anti-CD103-FITC)	FIT-22	Standard biotools	3160011C	100
161Dy	IFNAR-1	EP899Y	Abcam	213331	500
162Dy	CD11c	N418	Standard biotools	3162017C	100
163Dy	Anti-APC (anti-TNFa-APC)	APC003	Standard biotools	3163001C	100
164Dy	Bcl2	poly	R&D systems	AF810	1000
165Ho	CD95 (Fas)	Jo2	BD	554255	100
166Er	Eomes	dan11mag	Thermo Fisher Scientific	14-4875-82	100
167Er	NKp46/CD335	29A1.4	Standard biotools	3167008B	100
168Er	CD8a	53-6.7	Standard biotools	3168003B	500
169Tm	Sca1 (Ly6A/E)	D7	Standard biotools	3169015B	500
170Er	TCRgd	eBioGL3	Thermo Fisher Scientific	14-5711-82	120
171Yb	CD44	IM7	Standard biotools	3171003c	1500
172Yb	IL-2Rb (CD122)	TMB1	Biolegend	123223	100
173Yb	GzmB	GB11	Standard biotools	3173006B	1000
174Yb	LAG3/CD223	C9B7W	Standard biotools	3174019B	100
175Lu	IL7Ra/ CD127	A7R34	Standard biotools	3175006C	100
176Yb	ICOS	7E.17G9	Standard biotools	3176014B	200
209Bi	I-A/I-E (MHCII)	M5/114.15.2	Standard biotools	3209006C	4000
FITC	CD103-FITC	2E7	Biolegend	121419	100
PE	IFNg-PE	xMG1.2	Biolegend	505807	100
APC	TNFa-APC	MP6-XT22	Biolegend	506307	100

FIGURE 5D and Fig. S8C

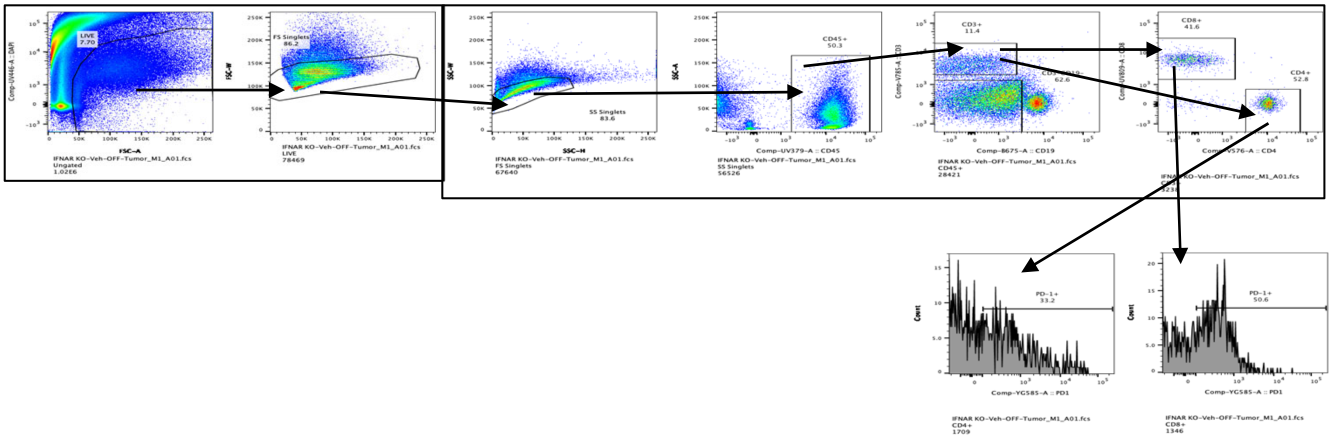

Fig2a, S4E,S7A

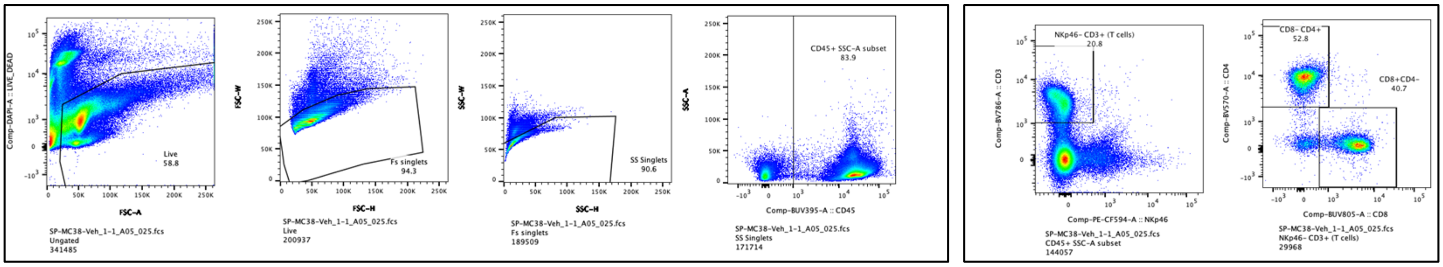

Supplemental Figure 18. Flow cytometry gating strategy

FIGURE 6B

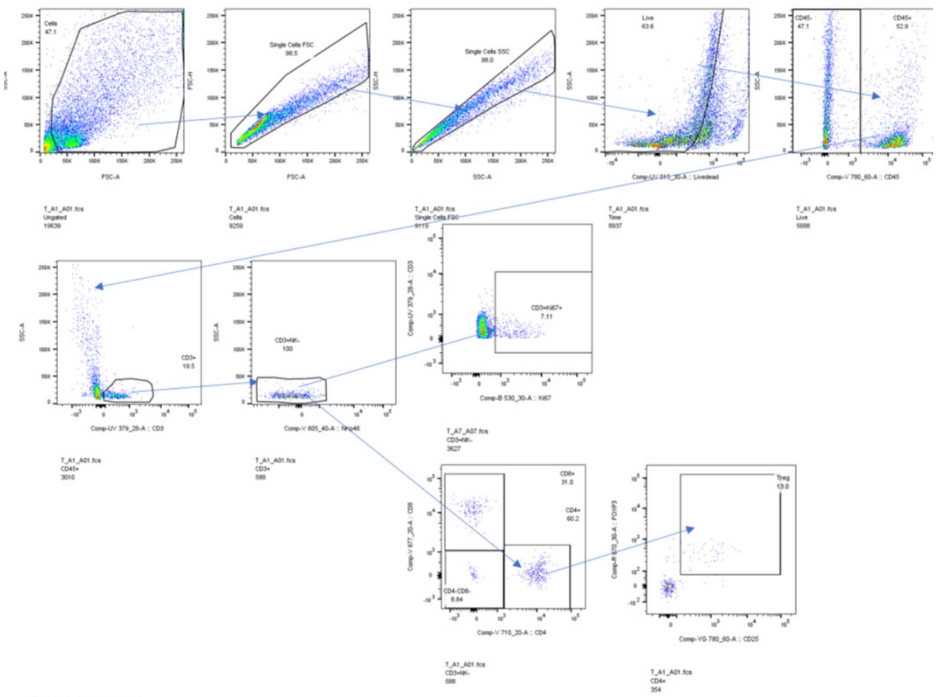

FIGURE 6B

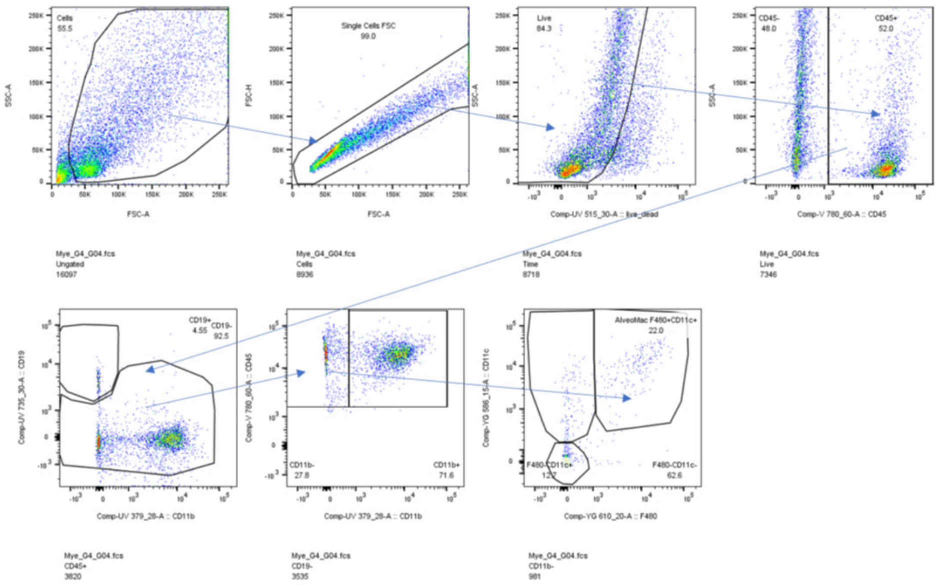

FIGURE S4D

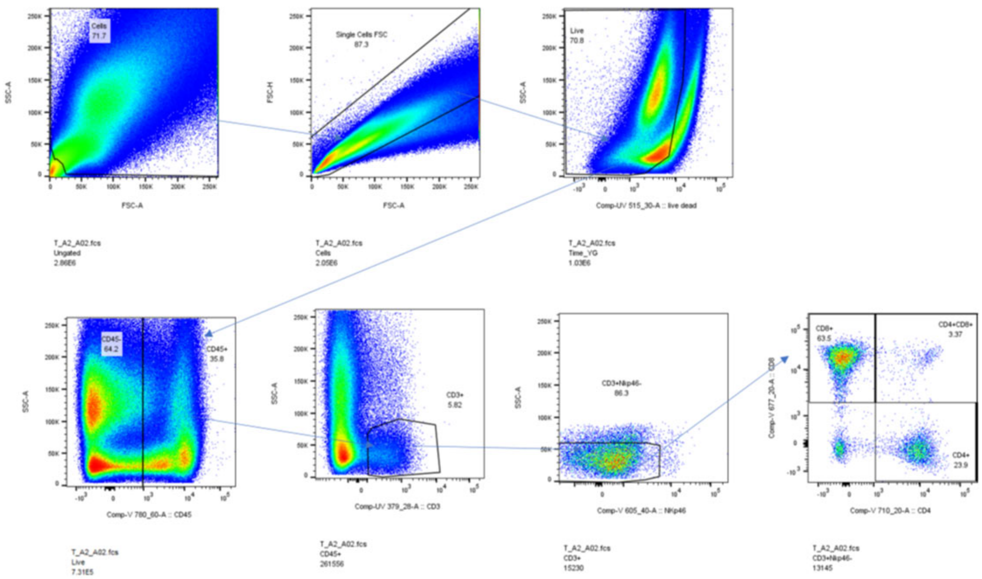

Supplemental Figure 18. Flow cytometry gating strategy

Flow cytometry gating strategy

FIGURE 5 panel C

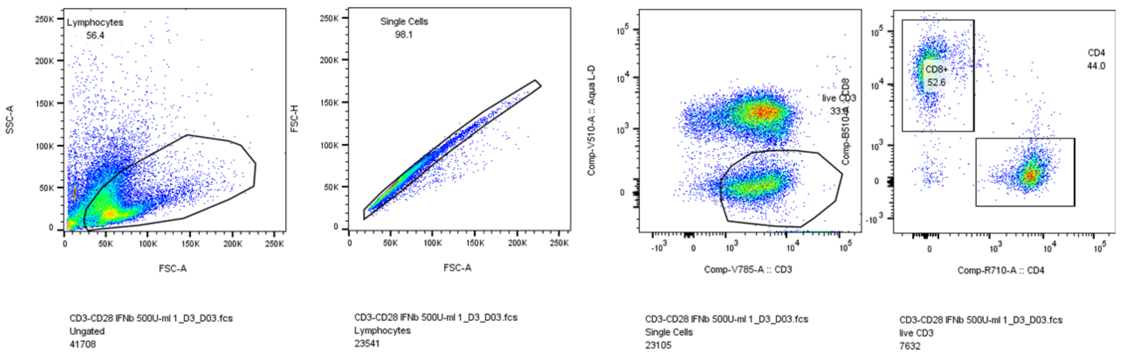

FIGURE 7 panel B

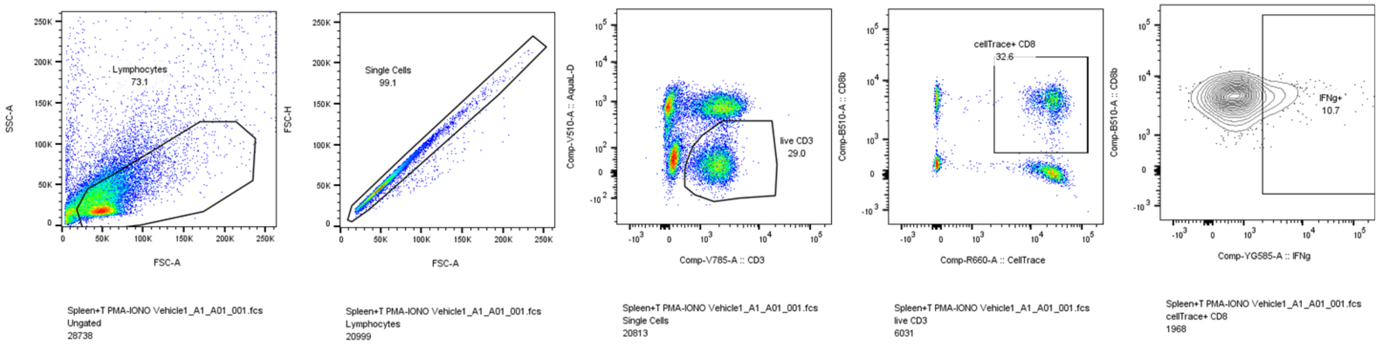

FIGURE 7 panel C,D

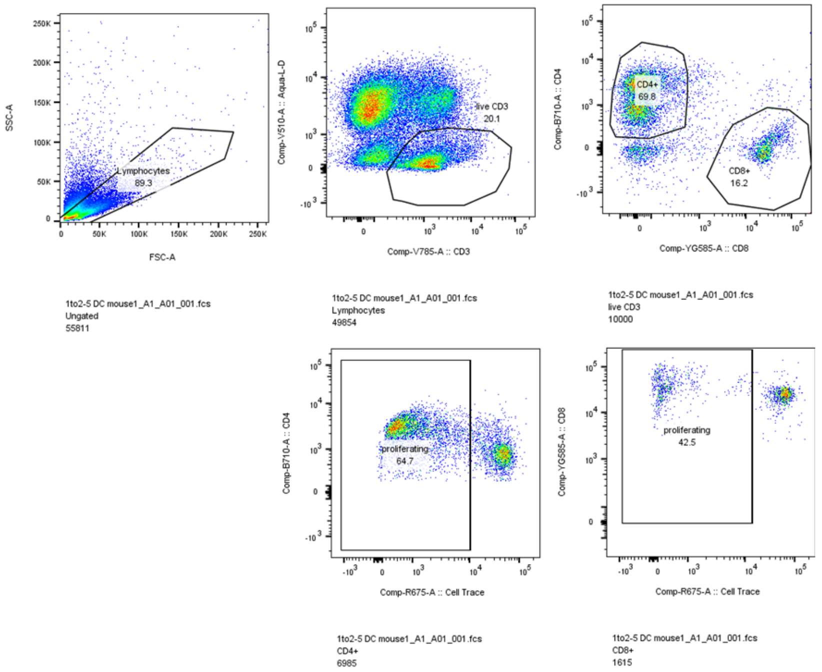

FIGURE 7 panel E

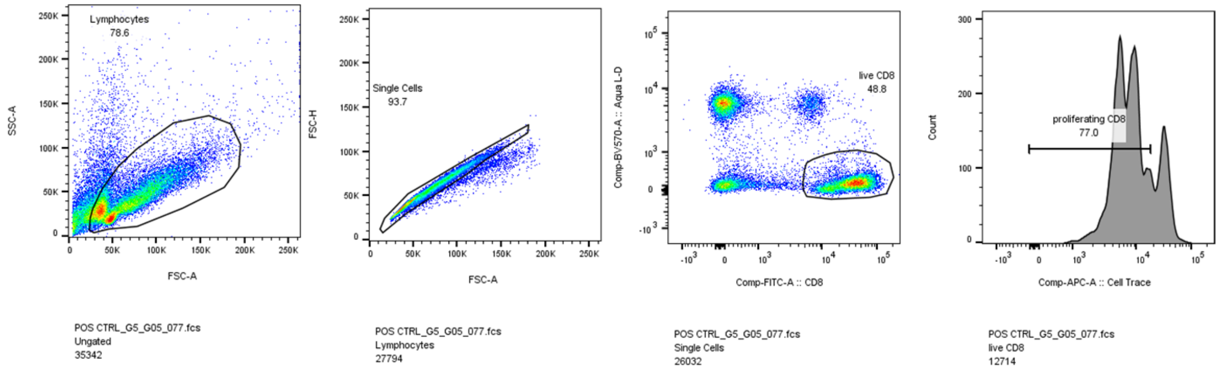

Supplemental Figure 18. Flow cytometry gating strategy

FIGURE S9 panel A

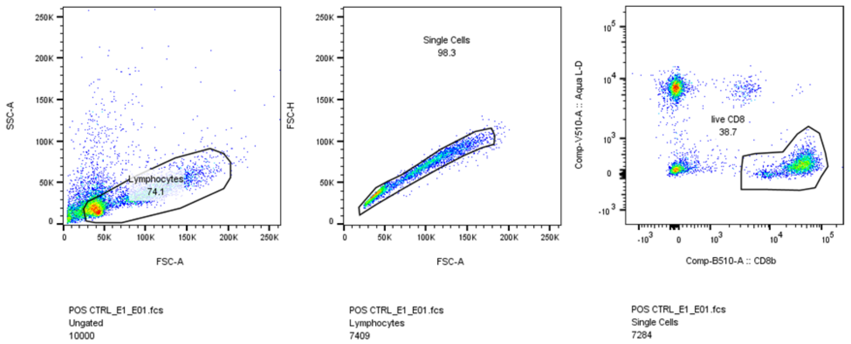

FIGURE S17 panel A and B

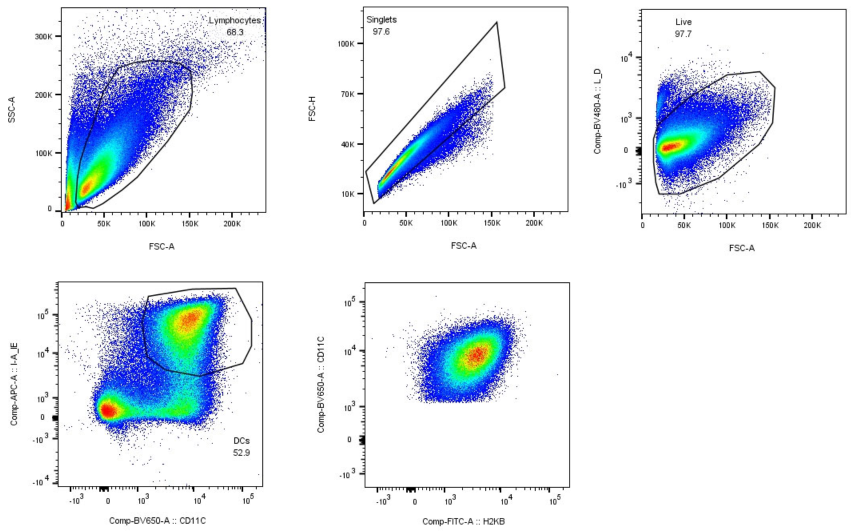

FIGURE S10 panels A and B

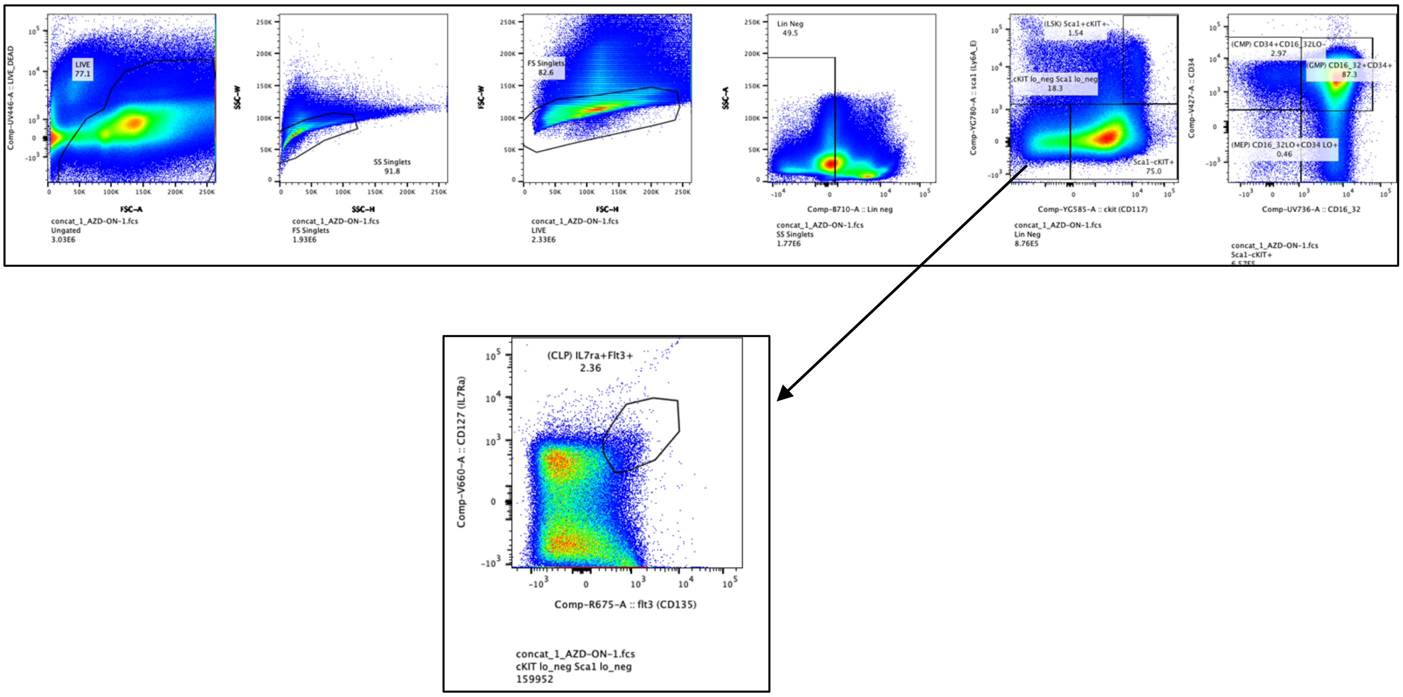

Supplemental Figure 18. Flow cytometry gating strategy

FIGURE S9 panels B and D

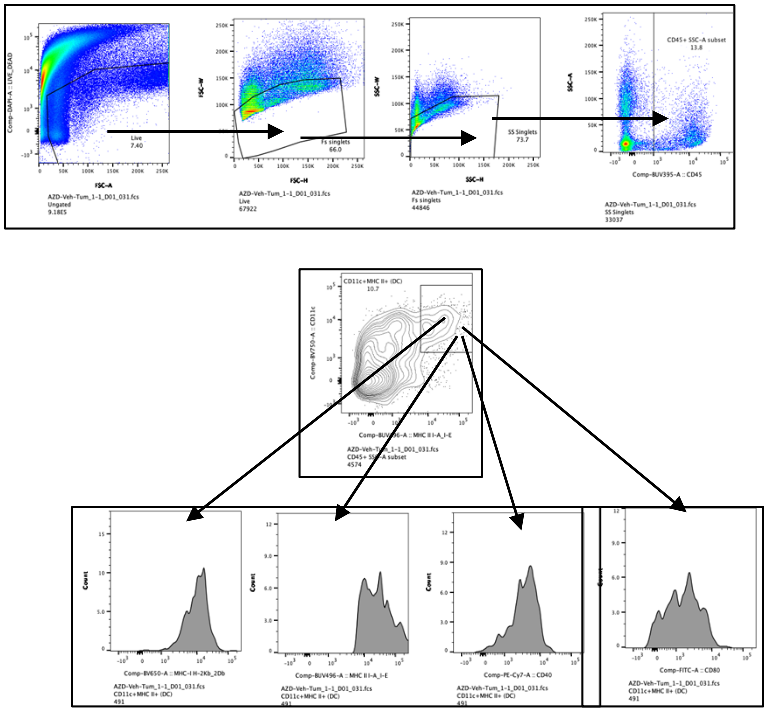

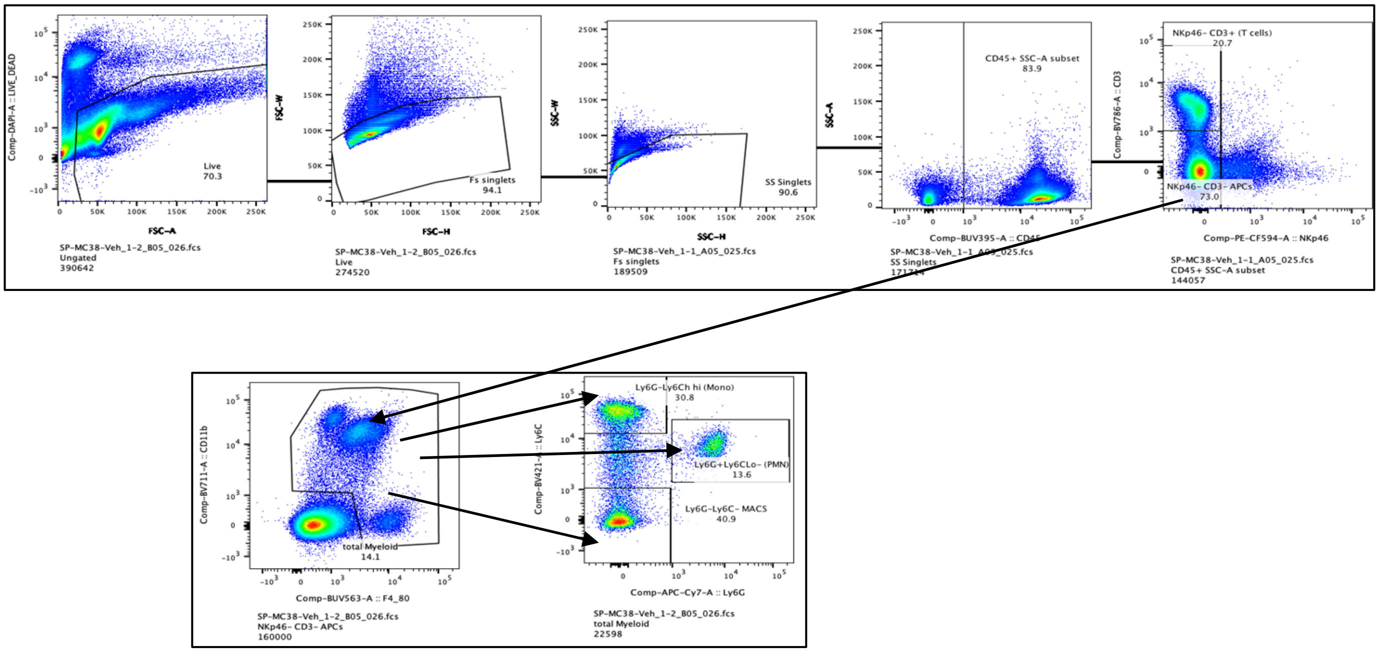

FIGURE S8 panels A and B

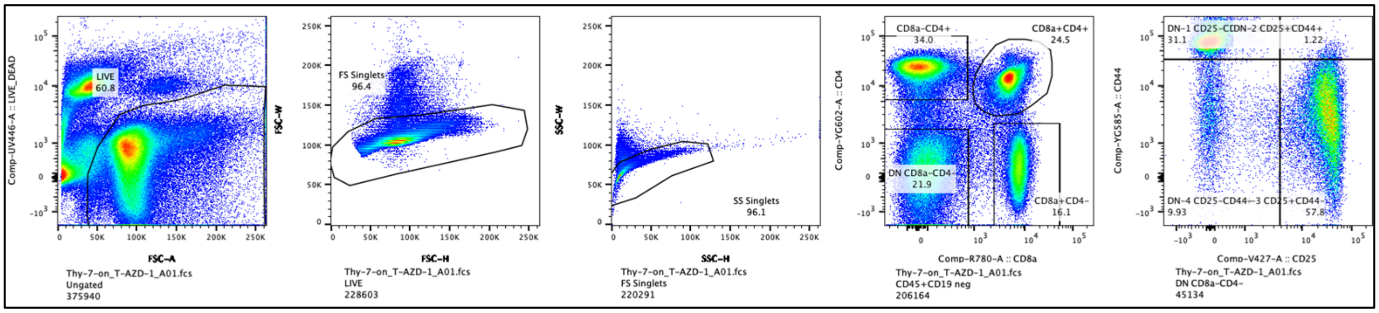

Supplemental Figure 18. Flow cytometry gating strategy
