# Supplementary material for: The ATR inhibitor ceralasertib potentiates cancer checkpoint immunotherapy by regulating the tumor microenvironment
Source: Nat Commun. 2024 Feb 24;15:1700. doi: 10.1038/s41467-024-45996-4 (PMC10894296; doi:10.1038/s41467-024-45996-4)
Supplement: Supplementary file 3 — Reporting Summary [file 41467_2024_45996_MOESM3_ESM.pdf]

nature portfolio

Corresponding author(s): Dmitry Gabrilovich

Last updated by author(s): Dec 21, 2023

Reporting Summary

Nature Portfolio wishes to improve the reproducibility of the work that we publish. This form provides structure for consistency and transparency in reporting. For further information on Nature Portfolio policies, see our Editorial Policies and the Editorial Policy Checklist.

Statistics

For all statistical analyses, confirm that the following items are present in the figure legend, table legend, main text, or Methods section.

n/a	Confirmed	
☐	☒	The exact sample size (*n*) for each experimental group/condition, given as a discrete number and unit of measurement
☐	☒	A statement on whether measurements were taken from distinct samples or whether the same sample was measured repeatedly
☐	☒	The statistical test(s) used AND whether they are one- or two-sided<br>*Only common tests should be described solely by name; describe more complex techniques in the Methods section.*
☐	☒	A description of all covariates tested
☐	☒	A description of any assumptions or corrections, such as tests of normality and adjustment for multiple comparisons
☐	☒	A full description of the statistical parameters including central tendency (e.g. means) or other basic estimates (e.g. regression coefficient) AND variation (e.g. standard deviation) or associated estimates of uncertainty (e.g. confidence intervals)
☐	☒	For null hypothesis testing, the test statistic (e.g. *F*, *t*, *r*) with confidence intervals, effect sizes, degrees of freedom and *P* value noted<br>*Give P values as exact values whenever suitable.*
☒	☐	For Bayesian analysis, information on the choice of priors and Markov chain Monte Carlo settings
☒	☐	For hierarchical and complex designs, identification of the appropriate level for tests and full reporting of outcomes
☒	☐	Estimates of effect sizes (e.g. Cohen's *d*, Pearson's *r*), indicating how they were calculated

Our web collection on statistics for biologists contains articles on many of the points above.

Software and code

Policy information about availability of computer code

Data collection	N/A
Data analysis	GraphPad Prism 6.0

For manuscripts utilizing custom algorithms or software that are central to the research but not yet described in published literature, software must be made available to editors and reviewers. We strongly encourage code deposition in a community repository (e.g. GitHub). See the Nature Portfolio guidelines for submitting code & software for further information.

Data

Policy information about availability of data

All manuscripts must include a data availability statement. This statement should provide the following information, where applicable:

- Accession codes, unique identifiers, or web links for publicly available datasets
- A description of any restrictions on data availability
- For clinical datasets or third party data, please ensure that the statement adheres to our policy

Raw data for sequencing are deposited Array express with links: https://www.ebi.ac.uk/biostudies/arrayexpress/studies/E-MTAB-13703 https://www.ebi.ac.uk/biostudies/arrayexpress/studies/E-MTAB-13704 https://www.ebi.ac.uk/biostudies/arrayexpress/studies/E-MTAB-13707 https://www.ebi.ac.uk/biostudies/arrayexpress/studies/E-MTAB-13713. Source data have been provided with this paper.

Human research participants

Policy information about studies involving human research participants and Sex and Gender in Research.

Reporting on sex and gender	Male 65.8%, Age <65 57% >65 43%
Population characteristics	21 patients were randomized: n=12 to ceralasertib and n=9 to Olaparib. Only ceralasertib data is presented in this manuscript. Two patients from ceralasertib arm were excluded due to protocol deviations. All patients provided informed consent for the analysis performed.
Recruitment	NCT03022409 (D5330C00007; Study 7) is an open-label, randomized window of opportunity trial where patients with head and neck squamous cell carcinoma (HNSCC) were treated with either the PARP inhibitor olaparib (300mg b.i.d) or ceralasertib (160mg b.i.d) for 9-21 days, prior to definitive surgery (or on-treatment biopsy) (Fig.S5)15.
Ethics oversight	The study is being performed in accordance with consensus ethical principles derived from international guidelines including the Declaration of Helsinki and Council for International Organizations of Medical Sciences (CIOMS) International Ethical Guidelines, applicable International Council for Harmonisation (ICH) Good Clinical Practice (GCP) Guidelines, all applicable laws and regulations, and the AstraZeneca policy on Bioethics and Human Biological Samples. The study protocol, protocol amendments, informed consent form, investigator's brochure, and other relevant documents were reviewed and approved by the Institutional Review Board/Ethics Committee at each participating centre.

Note that full information on the approval of the study protocol must also be provided in the manuscript.

Field-specific reporting

Please select the one below that is the best fit for your research. If you are not sure, read the appropriate sections before making your selection.

- [x] Life sciences
- [ ] Behavioural & social sciences
- [ ] Ecological, evolutionary & environmental sciences

For a reference copy of the document with all sections, see nature.com/documents/nr-reporting-summary-flat.pdf

Life sciences study design

All studies must disclose on these points even when the disclosure is negative.

Sample size	Each group had at least 10 mice. This number was chosen based on previous experience of therapeutic effect of immunoncology drugs.
Data exclusions	No data exclusions were perfromed
Replication	Each experiment (containng minimum of three biological replicates) was repeated at least three times to ascertain reproducibility
Randomization	Group allocations was random before implantation of tumors.
Blinding	Experiments were not blinded due to the nature of the experiments. However, experimental design, group assignment, and evaluation of the results were performed by different people not directly communicating with each other until the results are read.

Reporting for specific materials, systems and methods

We require information from authors about some types of materials, experimental systems and methods used in many studies. Here, indicate whether each material, system or method listed is relevant to your study. If you are not sure if a list item applies to your research, read the appropriate section before selecting a response.

Materials & experimental systems

n/a	Involved in the study	
[ ]	[x]	Antibodies
[ ]	[x]	Eukaryotic cell lines
[x]	[ ]	Palaeontology and archaeology
[ ]	[x]	Animals and other organisms
[x]	[ ]	Clinical data
[x]	[ ]	Dual use research of concern

Methods

n/a	Involved in the study	
[x]	[ ]	ChIP-seq
[ ]	[x]	Flow cytometry
[x]	[ ]	MRI-based neuroimaging

Antibodies

Antibodies used	Tables are provided in the manuscript
Validation	All data are provided in the manuscript.

Eukaryotic cell lines

Policy information about cell lines and Sex and Gender in Research

Cell line source(s)	All cell lines were obtained from ATCC
Authentication	Cell lines were not additionally authenticated
Mycoplasma contamination	All cell lines were regularly tested for mycoplasma contamination
Commonly misidentified lines (See ICLAC register)	Not used

Animals and other research organisms

Policy information about studies involving animals; ARRIVE guidelines recommended for reporting animal research, and Sex and Gender in Research

Laboratory animals	Female 8- to 10-week-old C57BL/6 and BALB/c mice were obtained from Envigo. Female 8- to 10-week-old IFNAR1 KO mice (B6(Cg)-Ifnar1tm1.2Ees/J), B6.SJL-Ptprca Pepcb/BoyJ were obtained from The Jackson Laboratory. For studies with bone marrow chimeras, B6.SJL-Ptprca Pepcb/BoyJ mice were irradiated at 1000 RAD and intravenously engrafted with 5x106 donor bone marrow cells isolated from either wild-type C57BL/6 mice, IFNAR1 KO (B6(Cg)-Ifnar1tm1.2Ees/J) or IFNAR1SA29. Mice were observed for 8 weeks for the full reconstitution of the immune compartment before the start of the experiment. Mice that achieved >90% chimerism were used for the study. In experiments with OdIN mice both male and female mice were used.
Wild animals	N/A
Reporting on sex	Experiments in transgenic mice were performed using mixed males and females. All other studies were performed in female mice. Sex was considered in study design and it was determined that female mice are better suited for proposed experiments.
Field-collected samples	N/A
Ethics oversight	Murine experiments in the US were approved by the Institutional Animal Care and Use Committee of AstraZeneca (Gaithersburg, MD) and conducted in Association for Assessment and Accreditation of Laboratory Animal Care (AAALAC)–accredited and United States Department of Agriculture (USDA)–licensed facility and the AstraZeneca Global Bioethics policy. Murine studies in the UK were conducted in accordance with UK Home Office legislation, the Animal Scientific Procedures Act 1986, the AstraZeneca Global Bioethics policy or Institutional Animal Care and Use Committee guidelines. Experimental work is outlined in project license PP3292652.

Note that full information on the approval of the study protocol must also be provided in the manuscript.

Flow Cytometry

Plots

Confirm that:

- [x] The axis labels state the marker and fluorochrome used (e.g. CD4-FITC).
- [x] The axis scales are clearly visible. Include numbers along axes only for bottom left plot of group (a 'group' is an analysis of identical markers).
- [x] All plots are contour plots with outliers or pseudocolor plots.
- [x] A numerical value for number of cells or percentage (with statistics) is provided.

Methodology

Sample preparation	Single cell suspension from disaggregated tumor, spleen and bone marrow cells were prepared and red blood cells lysed using ACK lysing buffer (Life Technologies). Cells were incubated with Fc-block for 10 minutes at 40C followed by staining with a cocktail of MAbs
Instrument	BD Symphony flow cytometer.
Software	Results were analyzed using FlowJo version 10.7.1 software.
Cell population abundance	Post-sort purity where applicable was >98%. Cell abundance was >10e5

Gating strategy	Gating strategy utilize FSC/SSC, cell aggregation exclusion, viability stain and the use of isotype controls for setting up the gates.

☒ Tick this box to confirm that a figure exemplifying the gating strategy is provided in the Supplementary Information.
